# Supplementary material for: Single molecule imaging simulations with advanced fluorophore photophysics
Source: Commun Biol. 2023 Jan 16;6:53. doi: 10.1038/s42003-023-04432-x (PMC9842740; doi:10.1038/s42003-023-04432-x)
Supplement: Supplementary file 2 — Supplementary Information [file 42003_2023_4432_MOESM2_ESM.pdf]

Supplementary Information

Single Molecule Imaging Simulations with Advanced Fluorophore Photophysics.

Dominique Bourgeois

Institut de Biologie Structurale, Université Grenoble Alpes, CNRS, CEA, IBS, 38044 Grenoble, France

Email : dominique.bourgeois@ibs.fr

Supplementary Note 1 (Application 1, Fig. 1)

Influence of pH

The reported pKa's of mEos4b in the green and red state are 5.5 and 5.8, respectively. Thus, at pH 8.5 the photoconversion efficiency is decreased and the red-state brightness is increased, while the opposite is true at pH 6.5. SMIS simulations suggest that at 3.5 kW/cm² better NPC images can be obtained at acidic pH. This is explained by the fact that at such readout power density the photon budget per localization is not decreased at moderately low pH (Supplementary Fig. S4c) because the median on-times are still significantly shorter than the frame time (Supplementary Fig. S4f). Strikingly, at 0.5 kW/cm² a reverse effect is observed: the image quality is better at pH 8.5. The explanation is that at pH 6.5 substantially more spots are missed (false negatives) during localization, and overall the degraded localization precision and density cannot be compensated for by the higher photoconversion efficiency.

Fermi profiles

Neglecting green-state photophysics, optimized Fermi profiles in principle allow maintaining a constant density of newly photoconverted red molecules along data collection. Their use permits to reduce the localization density (and therefore the numbers of spot overlaps) while maintaining the data collection time, or to reduce the data collection time while maintaining the localization density reached at constant 405-nm power. The price to pay, however, is a change in red-state photophysics along data collection resulting from the photosensitivity of dark states to 405-nm light[1]. Supplementary Fig. S5 shows that green-state photophysics compromise the possibility to maintain constant photoconversion when Fermi profiles are used, the effect being much more pronounced at 3.5 kW/cm² readout power density. Moreover, Panel 8 in Fig. 1b shows that the application of a Fermi profile while maintaining the data collection time (here 1200 s) is highly detrimental to the quality of the obtained NPC images. The reason is that with such profile (Supplementary Fig. S5e) photoconversion of most green mEos4b molecules by the 405-nm laser is delayed, while photobleaching by the 561-nm readout laser takes place at a constant rate. Hence, the photoconversion efficiency decreases substantially. This effect is much less pronounced at 0.5 kW/cm², and here there is a small gain in using a Fermi profile, attributed to the improved localization sparsity. Interestingly, the best NPC image is obtained at 3.5 kW/cm² using a Fermi profile with reduced data collection time. Here indeed, with no penalty in localization density, mEos4b molecules get photoconverted earlier, reducing green-state photobleaching and increasing the overall photoconversion efficiency. However, the data highlight that in all conditions tested, Fermi profiles result in substantially broader distributions of off-times in the mEos4b red state (Supplementary Fig. S4e), possibly compromising blinking correction algorithms employed for e.g. molecular counting.

Supplementary Note 2 (Application 2, Fig. 2)

Comparison of the photophysical behaviors of mEos4b and PAmCherry

Interestingly, it can be seen that, despite its green state being highly sensitive to photobleaching by 488 nm light, the performance of mEos4b as a non-priming partner is globally similar to that of PAmCherry (Fig. 2e),

which does not absorb cyan light in its non-activated state. This stems from the fact that, during the priming phase, in the absence of 405 nm light, mEos4b gets quickly shelved into its long-lived dark state, which largely protects it from photobleaching (Supplementary Fig. S8). Artificially removing in SMIS the capacity of green mEos4b to reversibly photoswitch not only results in major photobleaching during the priming phase, but also significantly increases spurious readout photoconversion during that phase, resulting in very poor colocalization (Fig. 2e and 2f). The SMIS generated datasets also highlight the impact of the different brightness of the simulated fluorescent proteins in the red states (Fig. 2d, Supplementary Fig. S6 and Supplementary Fig. S9). With a reported pKa of 7.9[2], a high fraction of the Dendra2 red chromophores remain protonated and nonfluorescent, reducing the measured photon budget per localization (Supplementary Fig. S9) and producing more blurry spots than mEos4b (Fig. 2d). With its lower intrinsic brightness as compared to mEos4b, the photon budget per localization of PAmCherry was also reduced, but this was somewhat compensated by a more complete photoactivation efficiency (Fig. 2f). Finally, as a result of their significantly different effective extinction coefficients, and therefore excitation rates by the 561 nm laser, it is interesting to notice the different levels of triplet state saturation observed in red mEos4b and Dendra2. Whereas there was almost no loss in photon budget due to intersystem crossing to T1 in the case of Dendra2 (~3%), a decrease of ~9% was noticed in the case of mEos4b (Supplementary Fig. S9).

Supplementary Note 3 (Application 3, Fig. 3)

Discrepancy between simulated and experimental dSTORM data

Although a clear decrease in apparent labeling efficiency is observed as the 647 nm laser power density is increased (Fig. 3e), in line with increasing saturation of the triplet state (Supplementary Fig. S11, Supplementary Fig. S12a) from which photobleaching occurs due photon reabsorption in T1, a significant decrease is also observed at the lowest power density, also seen at the ensemble level (Supplementary Fig. S12b). Also, while the number of photons per localization decreases at high power densities, as experimentally observed, it does not rise as high as expected at lower densities (Fig. 3f). Similarly, the number of localizations per fluorophore drops, as expected, at high densities but plateaus and even decreases at lower densities (Fig. 3e), and a similar trend occurs for the global photon budget per molecule (Supplementary Fig. S12d). The highest discrepancy between the simulated and experimental data concerns the on-times (Supplementary Fig. S12c) which tend to rise in SMIS simulations instead of globally decreasing in the experimental case as the 647 nm laser power density increases.

Discussion on the photophysical model of Cy5

To investigate the possible origin for partial disagreement between the SMIS simulations and the experimental data, we attempted to modify some of the interconversion rates and quantum yields in the Gidi et al model[3]. First, the decrease in labeling efficiency at low power observed in the SMIS data could be assigned to thermal recovery from the SR^- adduct state, which reduces the duty cycle of Cy5 switching at such low power. Thus, we assumed that in the *in cellulo* environmental conditions, the thermal recovery from SR- could be slower by a factor of ~4. Then, to increase the contrast in photon budget between low and high intensities, we reasoned that

the triplet state saturation could be higher than predicted by the Gidi et al model in the experimental data, for example due to a slightly underestimated pH or concentration of β-mercaptoethanol. We also assumed that the ~4-fold superior rate of adduct formation from the singlet state, as compared to the triplet state, measured by Gidi et al[3] could be significantly lower in cellular conditions. The modified interconversion rates and quantum yields (Supplementary Table S6) provided the titration data reported in Fig. 3e-f (dashed lines). A more consistent labeling efficiency at the lowest power density was retrieved (Fig. 3e), as well as a better qualitative trend in terms of localizations per fluorophore (Fig. 3e) and photon budget (Supplementary Fig. S12d). However, the number of photons per localization (Fig. 3f) and the on times (Supplementary Fig. S12c) still deviated from the experimental data.

We reasoned that if the main dark state responsible for adduct formation and photobleaching has a lifetime inferior to the shortest frametime in the titration series (which is the case for the μs-lived triplet state), a reduction of the on times as laser illumination is augmented can only be reached if T1 saturation is lower than ~50% at the highest intensity (Supplementary Note 1). Such a condition, however, would be incompatible with the high contrast observed throughout the titration series in effective labeling densities, photons per localization and localizations per fluorophore. Thus, we conclude that a long-lived (ms) dark state is likely involved in controlling Cy5 photobleaching in dSTORM experiments. In fact, hints towards the existence of such a state has been provided experimentally[4,5], and a very long-lived triplet state had to be invoked by Diekmann et al[6] to explain their data using a simple photophysical model. Yet, T1 cannot be ms-lived under dSTORM conditions. Another argument in disfavor of the μs-lived T1 being the pivotal dark state is the huge photobleaching quantum yield >> 10^{-3} required in SMIS to explain the experimental data. The long-lived dark state could possibly involve one of the anionic or cationic radical states, or be generated from the cis state of Cy5, but the conclusion is that, despite its sophistication, the model of Gidi et al still appears insufficient to explain the experimental dSTORM data. More complex schemes could be tested with SMIS, but the nature of the presumed long-lived dark state remains to be elucidated.

Supplementary Note 4 (Application 3, Fig. 3)

Estimation of the dependence of Cy5 on times as a function of laser power density

Here, we show that, using the Cy5 photophysical model of Gidi et al[3] with the short-lived triplet state as the main source of photobleaching , a decrease in on times as the laser power density increases is incompatible with a high contrast in the number of emitted fluorescence photons between high and low intensity lasers.

The rate of triplet state formation is given by: $k_{ST} = k_{exc}^{S} \times Q_{ISC}$, where k_{exc}^{S} is the excitation rate of the singlet state and Q_{ISC} is the quantum yield of intersystem crossing. The characteristic time spent in the singlet state before intersystem crossing is $\tau_S = 1/\, k_{ST}$. The rate for combined sulfur adduct formation and photobleaching from the triplet state is given by $k_{TSAB} = k_{TSA} + k_{exc}^{T} \times Q_B$ where k_{TSA} is the thermally induced rate of sulfur adduct formation, k_{exc}^{T} is the excitation rate of the triplet state and Q_B is the photobleaching

quantum yield from that state. The number of accesses to the triplet state to achieve a transition to either the sulfur adduct or the photo bleached state can then be estimated by:

$$N_T \approx 1/\tau_T \times 1/k_{TSAB} \qquad [1]$$

with τ_T being the triplet state lifetime. As $N_T \gg 1$ when τ_T is in the microsecond range, we can estimate the on-time in [s] as:

$$t_{ON}\ [s] \approx N_T \times (\tau_T + \tau_S) \qquad [2]$$

Now let's convert the on-time in units of frametimes, keeping in mind that frame times are adjusted to obtain a constant illumination dose. At a given laser power density, the frame time F can thus be written: $F\ [s] = K/k_{exc}^S$, where K is the number of excitation of the singlet state per frame. Therefore, the on-time in units of frametimes can be expressed as:

$$t_{ON}\ [f] \approx k_{exc}^S/K \times N_T \times (\tau_T + \tau_S) \qquad [3]$$

At low laser power density, there is little photobleaching from the triplet state and we have $k_{TSAB} \approx k_{TSA}$. Thus the on time $t_{ON}^L\ [f]$ reduce to:

$$t_{ON}^L\ [f] \approx k_{exc,L}^S/K \times 1/k_{TSA} \times \left(1 + {\tau_S^L}/{\tau_T}\right) \qquad [4]$$

with τ_S^L being the characteristic time before switching to the triplet state and $k_{exc,L}^S$ the excitation rate under low intensity illumination. In contrast, at the highest laser power density, photobleaching predominates so that $k_{exc}^T \times Q_B = \alpha \times k_{TSA}$ with $\alpha > 1$ (in the case of Diekmann et al[6], $\alpha \approx 3$ at 480 kW/cm²). Thus the on time $t_{ON}^H\ [f]$ is expressed as:

$$t_{ON}^H\ [f] \approx k_{exc,H}^S/K \times 1/(\alpha + 1)k_{TSA} \times \left(1 + {\tau_S^H}/{\tau_T}\right) \qquad [5]$$

Now the ratio of the on times at low and high laser eliminations is given by:

$$r_{L/H} = \frac{t_{ON}^L\ [f]}{t_{ON}^H\ [f]} \approx (\alpha + 1) \times \frac{k_{exc,L}^S \times \left(1 + {\tau_S^L}/{\tau_T}\right)}{k_{exc,H}^S \times \left(1 + {\tau_S^H}/{\tau_T}\right)} = (\alpha + 1) \times \frac{1 + {\tau_T}/{\tau_S^L}}{1 + {\tau_T}/{\tau_S^H}} \qquad [6]$$

At low intensity, we have ${\tau_T}/{\tau_S^L} \ll 1$, as there is low triplet state saturation. Thus, the expression above simplifies to:

$$r_{L/H} = (\alpha + 1) \times \frac{\tau_S^H}{\tau_S^H + \tau_T} \qquad [7]$$

For $r_{L/H} > 2$ and $\alpha \approx 3$, as observed experimentally, we arrive at the following constraint:

$$\tau_S^H > \tau_T \qquad [8]$$

This condition implies a rather low triplet state saturation of < 50% of the highest illumination power density, which is incompatible with the high contrast in photon detection between low and high laser intensities.

Supplementary Note 5 (Application 5, Fig. 5)

SMIS SPT data with a 5 ms frametime

Reducing the total frame time to 5 ms, the expected 2-state model was now retrieved with highest probability in the absence of fast exchange (Supplementary Fig. S17b). However, in the presence of fast exchange, a wrong 3-state model was again found, this time because the fast exchange rate becomes close to the inverse of the shortened frametime. Suppressing sensitivity of vbSPT to the fast exchange process required raising the rates to values as high as 100,000 s^{-1} (Supplementary Fig. S17b). Of note, with mEos3.2, at 5 ms frame time, the localization precision limits the accuracy of the slow diffusion coefficient retrieval and no improvement is obtained by either of the techniques presented above (Supplementary Fig. S18-19).

Supplementary Table S1

Phototransformation quantum yields and thermal rates used for mEos4b in NPC SMIS simulations (Application 1)

	Green anionic	***Green neutral***	***Green short-lived dark***	***Green long-lived dark***	***Green bleached***	***Red anionic***	***Red neutral***	***Red short-lived dark***	***Red long-lived dark***	***Red bleached***
Green anionic		Rapid Exchange	3.5×10^{-5}	5.0×10^{-5}	2.5×10^{-6}	5×10^{-7}	0	0	0	0
Green neutral	Rapid Exchange		0	0	2.5×10^{-6}	1.0×10^{-4}	0	0	0	0
Green short-lived dark	1×10^{-3} ($0.1\ s^{-1}$)*	0		0	2.5×10^{-6}	0	0	0	0	0
Green long-lived dark	1×10^{-2} ($0.001\ s^{-1}$)	0	0		2.5×10^{-6}	0	0	0	0	0
Red anionic							Rapid Exchange	5.0×10^{-6}	1.5×10^{-5}	1.0×10^{-5}
Red neutral						Rapid Exchange		0	0	1.0×10^{-5}
Red short-lived dark						1.0×10^{-4} ($20\ s^{-1}$)	0		0	1.0×10^{-5}
Red long-lived dark						5.0×10^{-3} ($0.001\ s^{-1}$)	0	0		1.0×10^{-5}

* Value in parentheses refer to thermally induced relaxation rates

Supplementary Table S2

Main parameters used in SMIS simulations (Application 1)

Simulation name	***Reference***	***3.5 kW/cm², pH 7.5***	***0.5 kW/cm², pH 7.5***	***3.5 kW/cm², pH 8.5***	***3.5 kW/cm², pH 6.5***	***0.5 kW/cm², pH 8.5***	***0.5 kW/cm², pH 6.5***	***3.5 kW/cm², long Fermi***	***0.5 kW/cm², long Fermi***	***3.5 kW/cm², short Fermi***
Frame time *(ms)*	50									
Number of frames	25000									8000
561-nm laser power density *[kW/cm²]*	3.5	3.5	0.5	3.5	3.5	0.5	0.5	3.5	0.5	3.5
405-nm laser power density *[kW/cm²]*	0.002							100[£]<br>2.7[*]<br>1200[$]	100[£]<br>6.7[*]<br>1200[$]	100[£]<br>5.3[*]<br>360[$]
Pixel size *[nm]*	125									
Emission filter band path *[nm]*	620 ± 42.5									
EMCCD gain	200									
EMCCD quantum efficiency	0.9									
EMCCD readout noise *[e⁻]*	74									
Objective numerical aperture	1.49									
mEos4b pH	7.5	7.5	7.5	8.5	6.5	8.5	6.5	7.5	7.5	7.5

£: Maximum power density
*: Fermi profile sharpness [s]
$: Fermi profile length [s]

Supplementary Table S3

Phototransformation quantum yields and thermal rates used for Dendra2 in primed photoconversion SMIS simulations (Application 2)

	Green anionic	***Green neutral***	***Green T1***	***Green short-lived dark***	***Green long-lived dark***	***Green bleached***	***Red anionic***	***Red neutral***	***Red T1***	***Red short-lived dark***	***Red long-lived dark***	***Red bleached***
Green anionic		Rapid Exchange	1.0×10^{-3}	3.5×10^{-5}	5.3×10^{-5}	1.0×10^{-5}	5×10^{-7}	0	0	0	0	0
Green neutral	Rapid Exchange		1.0×10^{-3}	0	0	1.0×10^{-5}	1.0×10^{-4}	0	0	0	0	0
Green T1	(2500 s^{-1})	0		0	0	0	1.0×10^{-3}	0	0	0	0	0
Green short-lived dark	1×10^{-3} (0.1 s^{-1})	0			0	1.0×10^{-5}	0	0	0	0	0	0
Green long-lived dark	1×10^{-1} (0.001 s^{-1})	0		0		1.0×10^{-5}	0	0	0	0	0	0
Red anionic								Rapid Exchange	1.0×10^{-3}	5.2×10^{-6}	8.8×10^{-6}	5.0×10^{-5}
Red neutral							Rapid Exchange		1.0×10^{-3}	0	0	0
Red T1							(2500 s^{-1})	0		0	0	0
Red short-lived dark							1.0×10^{-4} (16 s^{-1})	0	0		0	5.0×10^{-5}
Red long-lived dark							5.0×10^{-3} (0.001 s^{-1})	0	0	0		5.0×10^{-5}

* Value in parentheses refer to thermally induced relaxation rates

Supplementary Table S4

Main parameters used in SMIS simulations (Application 2)

Data collection phase	**Priming**	**UV**
Frame time *(ms)*	50	
Number of frames	10000	10000
561-nm laser power density *[kW/cm²]*	1	1
405-nm laser power density *[kW/cm²]*	0	0.005
488-nm laser power density *[kW/cm²]*	0.004	0
730-nm laser power density *[kW/cm²]*	0.5	0
Pixel size *[nm]*	100	
Emission filter band path *[nm]*	600 ± 20	
EMCCD gain	300	
EMCCD quantum efficiency	0.9	
EMCCD readout noise *[e^-]*	74	
Objective numerical aperture	1.49	
pH	7.5	

£: Maximum power density
*: Fermi profile sharpness
$: Fermi profile length

Supplementary Table S5

Phototransformation quantum yields and thermal rates used for Alexa647 (Application 3)

	Fluorescent state	*T1*	*Cationic radical*	*Anionic radical*	*Sulfur adduct*	*Bleached*
Fluorescent state		**7.1×10^{-4}** $^{\$}$	0	0	**3.2×10^{-6}**	5×10^{-7}
T1	**(3.5×10^{5} s^{-1})**		1.0×10^{-3}	(1000 s^{-1})	**(350 s^{-1})**	5.0×10^{-3}
Cationic radical	1×10^{-4}+(**2300 s^{-1}**)*	0		0	0	1.0×10^{-2}
Anionic radical	1×10^{-4}+(**1170 s^{-1}**)	0	0		0	1.0×10^{-2}
Sulfur adduct	**0.1**$^{£}$+(**0.01 s^{-1}**)					1.0×10^{-5}

*: Values in parentheses refer to thermally induced relaxation rates
$: Values in bold were extracted from Gidi et al[3]
£: Value calculated so that the product q×ε matches the action spectrum of Fig 4b in Gidi et al[3]

Supplementary Table S6

Phototransformation quantum yields and thermal rates used for Alexa647 (Application 3, modified parameters)

	Fluorescent state	*T1*	*Cationic radical*	*Anionic radical*	*Sulfur adduct*	*Bleached*
Fluorescent state		**7.1×10^{-4}** $^{\$}$	0	0	**3.2×10^{-7}** $^{\%}$	3.5×10^{-7}
T1 $^{\&}$	**(1.8×10^{5} s^{-1})**		1.0×10^{-3}	(1000 s^{-1})	**(867 s^{-1})**	4.0×10^{-3}
Cationic radical $^{\&}$	1×10^{-4}+(**2300 s^{-1}**)*	0		0	0	1.0×10^{-4}
Anionic radical $^{\&}$	1×10^{-4}+(**1170 s^{-1}**)	0	0		0	1.0×10^{-2}
Sulfur adduct $^{\&}$	**0.1**$^{£}$+(0.0025 s^{-1})					1.0×10^{-5}

&: Quantum yields are only indicative, as phototransformation rates are dictated by the product q×ε were ε is the associated extinction coefficient at the considered wavelength.
*: Values in parentheses refer to thermally induced relaxation rates
$: Values in bold were extracted from Gidi et al[3]
£: Value calculated so that the product q×ε matches the action spectrum of Fig 4b in Gidi et al[3]
%: Underlined values were adjusted from Gidi et al[3] to improve matching with experimental NPC dSTORM data

Supplementary Table S7

Extinction coefficients of Alexa647 (Application 3)

	Fluorescent state	*T1*	*Cationic radical*	*Anionic radical*	*Sulfur adduct*
$\varepsilon_{647\ nm}$ [$M^{-1}\times cm^{-1}$]	270000	71.5	2.75	0.09	0.0017
$\varepsilon_{405\ nm}$ [$M^{-1}\times cm^{-1}$]	0	0	39240	5057	400

Supplementary Table S8

Bleaching brightness of Alexa647 (Application 3)

	Fluorescent state	*T1*	*Cationic radical*	*Anionic radical*	*Sulfur adduct*
$q\times\varepsilon_{647\ nm}$ [$M^{-1}\times cm^{-1}$]	0.135	0.357	0.027	0.001	0
$q\times\varepsilon_{405\ nm}$ [$M^{-1}\times cm^{-1}$]	0	0	392 [&]	51 [&]	0.004

&: Photobleaching from the cationic and anionic radicals was deliberately set to high values to investigate the possible roles of these states in accelerated photobleaching. However, due to the very low level of 405 nm light used throughout data collection, the effective fraction of molecules that bleached from those states remained low (Supplementary Fig. S11)

Supplementary Table S9

Main parameters used in SMIS simulations (Application 3)

647-nm laser power density *[kW/cm²]*	**0.36**	**1.6**	**6.4**	**24**	**53**	**120**	**480**
Frame time *(ms)*	*500*	*110*	*28*	*7.5*	*3.5*	*1.5*	*0.38*
405-nm laser power density *[W/cm²] §*	0.01£<br>125*<br>7500$	0.05£<br>27.5<br>1650$	0.2£<br>7*<br>420$	0.77£<br>1.87*<br>112.5$	1.65£<br>0.87*<br>52.5$	3.75£<br>0.37*<br>22.5$	15£<br>0.09*<br>5.7$
Number of frames	25000						
Pixel size *[nm]*	125						
Emission filter band path *[nm]*	700 ± 40						
EMCCD gain	200						
EMCCD quantum efficiency	0.9						
EMCCD readout noise *[e^-]*	74						
Objective numerical aperture	1.49						
Objective transmission efficiency [%]	25.8						
Microscope optics transmission efficiency [%]	80						

§: Fermi profiles were applied for the 405 nm laser to keep the density of activated molecules approximately constant during data acquisition.
£: Maximum power density
*: Fermi profile sharpness [s]
$: Fermi profile length [s]

Supplementary Table S10

Phototransformation quantum yields and thermal rates used for Alexa647, CF680 and CF660C in Spectral Demixing SMIS simulations (Application 4)

	Fluorescent state		***Short-lived dark***	***Sulfur adduct***	***Bleached***
Fluorescent state			7.0×10^{-5}	1.0×10^{-7}	8.0×10^{-7}
Short-lived dark	0	(500 s^{-1})*		(50 s^{-1})*	7.0×10^{-5}
Sulfur adduct	0.1	(0.0025 s^{-1})*	0		0

* Value in parentheses refer to thermally induced relaxation rates

Supplementary Table S11

Main parameters used in SMIS simulations (Application 4)

Simulation name	***3-color AF647/CF660C/CF680***	***2-color AF647/CF660C***	***2-color AF647/CF680***	***2-color AF647/AF700***	***1-color AF647,CF660C or CF680***
Frame time *[ms]*	20				
Number of frames	10000 (25000$^{\$}$)				
Number of frames	*10,000*	10000<br>25000$^{\$}$	*10,000*		
561-nm laser power density *[W/cm^2] £*	2000-				
405-nm laser power density *[W/cm^2] £*	0.3	0.3<br>0.01$^{\$}$<br>ramp 0.75-15$^{\&}$	0.3		
Pixel size *[nm]*	100				
Dichroic filter	CHROMA T685LPXR			SEMROCK FF695-Di01	CHROMA T685LPXR
Emission filter Channel 1	CHROMA BPET68570			CAIRN BP68740	CHROMA BPET68570
Emission filter Channel 2	None				
Channel 2 chromatic distortion	[0.5, 0.7, 0.3, 0.2]$^{£}$			[0.7, 0.9, 0.4, 0.3]$^{£}$	[0.5, 0.7, 0.3, 0.2]$^{£}$
Fluorescence background *[ph/μm^2/W/cm^2/s]*	100	100<br>10$^{\#}$<br>500*	100		
EMCCD gain	200				
EMCCD quantum efficiency	0.9				
EMCCD readout noise *[e$^-$]*	74				
Objective numerical aperture	1.49				

$: Low-density dataset
&: High density dataset
£: X-shift [raster], Y-shift [raster], X-stretch [%], Y-stretch [%]
#: Low-background dataset
*: High-background dataset

Supplementary Table S12

Phototransformation quantum yields used for mEos3.2 in SPT SMIS simulations (Application 5)

	Green anionic	*Green neutral*	*Green short-lived dark*	*Green long-lived dark*	*Green bleached*	*Red anionic*	*Red neutral*	*Red short-lived dark*	*Red long-lived dark*	*Red bleached*
Green anionic		Rapid Exchange	3.5×10^{-5}	5.0×10^{-5}	2.5×10^{-6}	5×10^{-7}	0	0	0	0
Green neutral	Rapid Exchange		0	0	2.5×10^{-6}	1.0×10^{-4}	0	0	0	0
Green short-lived dark	1×10^{-3} ($0.1\ s^{-1}$)*	0		0	2.5×10^{-6}	0	0	0	0	0
Green long-lived dark	1×10^{-2} ($0.001\ s^{-1}$)	0	0		2.5×10^{-6}	0	0	0	0	0
Red anionic							Rapid Exchange	1.5×10^{-5}	4.5×10^{-5}	3.0×10^{-5}
Red neutral						Rapid Exchange		0	0	3.0×10^{-5}
Red short-lived dark						1.0×10^{-4} ($20\ s^{-1}$)	0		0	3.0×10^{-5}
Red long-lived dark						2.0×10^{-2} ($0.001\ s^{-1}$)	0	0		3.0×10^{-5}

* Value in parentheses refer to thermally induced relaxation rates

Supplementary Table S13

Phototransformation quantum yields used for PA-JF549 (Application 5)

	Off	*Fluorescent*	*Dark*	*Bleached*
Off &		0.02	0	0
Fluorescent	0		4.5×10^{-6}	3.0×10^{-6}
Dark &	0	0.02 ($35\ s^{-1}$)*		2.0×10^{-6}

&: Quantum yields are only indicative, as phototransformation rates are dictated by the product $q\times\varepsilon$ were ε is the associated extinction coefficient at the considered wavelength.
*: Values in parentheses refer to thermally induced relaxation rates

Supplementary Table S14

Main parameters used in SMIS simulations (Application 5)

Simulation name	***mEos3.2 -488 nm***	***mEos3.2 +488 nm***	***PA-JF549***	***mEos3.2 -488 nm***	***mEos3.2 +488 nm***	***PA-JF549***
Frame time *(ms)* &	5* (10)$			1* (4)$		
Number of frames	10000					
561-nm laser power density *[W/cm²]* £	750		-	3750		-
555-nm laser power density *[W/cm²]* £	-		750	-		3750
405-nm laser power density *[W/cm²]* £	2.1		0.04	5.25		0.1
488-nm laser power density *[W/cm²]* £	0	10	-	0	25	-
Pixel size *[nm]*	125					
Emission filter band path *[nm]*	650 ± 85					
EMCCD gain	300					
EMCCD quantum efficiency	0.9					
EMCCD readout noise *[e⁻]*	74					
Objective numerical aperture	1.49					
Objective depth of focus [nm]	500					

&: Stroboscopic mode
£: Maximum power density, flat beam profile
*: Duration of CCD/561-nm or 555-nm laser exposure
$: Duration of dark time between frames (405-nm and/or 488-nm laser exposure)

Supplementary Table S15

Available SMLM simulators

	Main purpose	Fluorophore description: Photophysical model	Spectra	SptPALM	3D	Multicolor	PSF	Lasers	Strength	Platform
SuReSim (ref [7])	SMLM design and validation	3 state	No	No	Yes	No	Scalar, 2D Gaussian, 3D Astigmatism	No	Speed; Advanced labeling	Java
SMeagol (ref [8])	Single particle tracking	Various predefined models	No	Yes	Yes	No	Scalar, 2D Gibson-Lanni	No	Advanced diffusion models	Matlab
TestSTORM (ref [9])	SMLM design and validation	3-state	Emission wavelength	No	Yes	2 color	Scalar/ Vectorial; 2D, 3D Astigmatism	No	Versatility, 2 color with crosstalk, polarization sensitivity	Matlab
(SR FightClub) (ref [10])	Input for testing localization software	4-state	No	No	Yes	No	Experimental 2D or 3D Astigmatism, Bi-plane, Double helix	No	Realistic spot shape, spot density and signal-to-noise ratio	Java
FluoSim (ref [11])	Simulating protein dynamics	2-state	No	Yes	No	No	Scalar, 2D Gaussian	No	Various modalities: SMLM, sptPALM, FRAP, FCS	C++
VirtualSMLM (ref [12])	Real-time SMLM simulations	4-state	No	No	Yes	No	Experimental 2D or 3D Astigmatism, Bi-plane, Double helix	Yes	Speed; Imaging parameters can be changed on the fly	Java
sptPALMsim (ref [13])	Single particle tracking	3-state	No	Yes	Yes	No	3D, pupil function model	No	Multiple diffusing states	Python
SMIS (This work)	Advanced fluorophore photophysics	N-state	Full spectra	Yes	Yes	Multicolor	Scalar, 2D Gaussian, 3D Astigmatism,	Yes	Same input parameters as on real microscope	Matlab

Supplementary Table S16

List of main SMIS features.

FLUOROPHORES:

- UNLIMITED NUMBER OF FLUOROPHORES (COLORS)
- NUMBER OF MOLECULES PER COLOR
- FRACTIONAL DISTRIBUTION ON VIRTUAL SAMPLE PATTERNS
- MATURATION LEVEL, LABELING EFFICIENCY
- MINIMUM DISTANCE BETWEEN FLUOROPHORES
- LINKAGE ERROR OPTION
- FLUOROGENICITY OPTION
- PH SENSITIVITY OPTION FOR FLUORESCENT STATES (PKA, HILL COEFFICIENT)
- DIPOLE ORIENTATION
  - FIXED OR TUMBLING, REORIENTATION RATE OPTION
- PHOTOPHYSICAL STATES
  - UNLIMITED NUMBER OF PHOTOPHYSICAL STATES
  - FLUORESCENCE QUANTUM YIELD FOR FLUORESCENT STATES
  - ABSORPTION, EXCITATION AND FLUORESCENCE EMISSION SPECTRA
  - THERMAL EXCHANGE RATES BETWEEN PHOTOPHYSICAL STATES
  - PHOTOTRANSFORMATION QUANTUM YIELDS BETWEEN PHOTOPHYSICAL STATES
- DIFFUSION BEHAVIOR
  - UNLIMITED NUMBER OF DIFFUSION STATES
  - DIFFUSION COEFFICIENTS
  - CONFINED DIFFUSION ON DEFINED VIRTUAL SAMPLE PATTERNS
  - DIRECTED MOTION OPTION
  - EXCHANGE RATE BETWEEN DIFFUSION STATES
- QUANTITATIVE PALM
  - DEFINED OLIGOMERIC STATE
- FLUOROPHORE PAIRING OPTION FOR COLOCALIZATION
- FRET OPTION BETWEEN PAIRS OF FLUOROPHORES

MICROSCOPE DESCRIPTION

- MICROSCOPE OBJECTIVE AND POINT SPREAD FUNCTION
  - NUMERICAL APERTURE
  - OBJECTIVE REFRACTIVE INDEX
  - SAMPLE REFRACTIVE INDEX
  - OBJECTIVE AND MICROSCOPE TRANSMISSION EFFICIENCIES
  - 2D OPTIONS
    - GAUSSIAN PSF
  - 3D OPTIONS
    - DEPTH OF FOCUS
    - 3D SAMPLE POSITIONING
    - GAUSSIAN OR ASTIGMATIC PSF
    - TIRF OR HILO MODES
- LASERS

- UNLIMITED NUMBER OF LASERS
- LASER DESCRIPTION:
  - WAVELENGTH
  - PROFILE (GAUSSIAN OR FLAT)
  - *NOMINAL POWER OR POWER DENSITY AT BEAM CENTER*
  - *INTENSITY ALONG DATA ACQUISITION (CONSTANT, RAMP, FERMI PROFILE, CUSTOM PROFILE)*
  - *POLARIZATION (CIRCULAR OR LINEAR)*
  - *TIMING (ON DURING FRAMES OR BETWEEN FRAMES)*
  - *FRAP MODE (LOCAL ILLUMINATION)*

- ACQUISITION CHANNELS AND FILTERS
  - 2-CHANNEL OPTION
    - DUAL CAMERA OR SPLIT SINGLE CAMERA
    - CHANNEL 2 DEFOCUS AND DEFORMATION OPTIONS
  - EMISSION AND DICHROIC FILTERS
    - EXPERIMENTAL FILTERS OPTION
- EMCCD CAMERA
  - EMCCD GAIN, QUANTUM EFFICIENCY, DYNAMIC RANGE, ADU OFFSET, READOUT NOISE, CLOCK - INDUCED CHARGE, CONVERSION FACTOR (e^-/ph, e^-/ADU)

DATA COLLECTION PARAMETERS

- FRAME SIZE, NUMBER OF FRAMES, PIXEL SIZE, FRAME TIME, TIME BETWEEN FRAMES
- SAMPLE DRIFT OPTION

SMIS TOOLS

- CREATE FLUOROPHORE TOOL
- CREATE VIRTUAL SAMPLE TOOL
- ANALYZE RESULTS TOOL

GENERAL SMIS PARAMETERS

- PARALLEL COMPUTING OPTION
- ENSEMBLE SIMULATION OPTION
- DATA ACQUISITION MONITORING
- PSF, FLUOROPHORE SPECTRA AND LASERS MONITORING TOOLS
- SAMPLING RATE OPTIMIZATION OPTION
- SPTPALM OPTIONS (SUB-FRAME DIFFUSION, DIFFUSION SAMPLING RATE)

Supplementary Table S17

Examples of available fluorophores in SMIS. See also Methods section for modifying available fluorophores or defining new fluorophores.

	Type	*Photophysical model*	*Spectra*	*pH dependence*	*Comment**
EGFP	FP	3-state	FPbase$	No	Basic, 1 dark state
EYFP	FP	3-state	FPbase	No	Basic, 1 dark state
mVenus	FP	2-state	FPbase	No	Basic, no blinking
mTurquoise2	FP	2-state	FPbase	No	Basic, no blinking
rsEGFP2	RSFP	5-state	In-house (ref [14])	No	Includes blinking and switching
rsEGFP2 cryo	RSFP	8-state	In-house (ref [15])	No	2 fluorescent states with corresponding blinking and bleaching
rsTagRFP	RSFP	4-state	ref [16]	No	Includes blinking and switching
Simple PCFP	PCFP	6-state	Based on ref [17]	Yes	Generic PCFP without blinking
mEos4b	PCFP	10-state	In-house (ref [17])	Yes	Includes blinking, switching and photoconversion
mEos4b T1	PCFP	11-state	In-house (ref [17])	Yes	Includes triplet state in the red state in addition to mEos4b model
mEos3.2	PCFP	10-state	FPbase +In-house (ref [17])	Yes	Includes blinking, switching and photoconversion
Dendra2 T1	PCFP	12-state	In-house (ref [18])	Yes	Includes blinking, switching, photoconversion and triplet state in both colors
PAmCherry1	PAFP	7-state	FPbase	Yes	Includes blinking and switching in red state
PAmCherry1 T1	PAFP	8-state	FPbase	Yes	Includes triplet state, blinking and switching in red state
Alexa647 STORM	Cyanine dye	4-state	SpectraViewer£	No	Ad hoc model for STORM: includes ms-lived and long-lived dark states
Alexa700 STORM	Near-IR dye	4-state	SpectraViewer	No	Ad hoc model for STORM: includes ms-lived and long-lived dark states
CF680 STORM	Cyanine dye	4-state	AAT Spectrum Viewer%	No	Ad hoc model for STORM: includes ms-lived and long-lived dark states
CF660 STORM	Cyanine dye	4-state	AAT Spectrum Viewer	No	Ad hoc model for STORM: includes ms-lived and long-lived dark states
Cy5 full	Cyanine dye	7-state	SpectraViewer and ref [3]	No	Full Cy5 model including triplet state, cis-state, anionic and cationic radicals and sulfur adduct

Atto647N PBS	Pyronine dye	5-state	ATTO-TEC	No	Ad-hoc model for Atto647N in PBS
Atto647N GLOX	Pyronine dye	5-state	ATTO-TEC	No	Ad-hoc model for Atto647N in deoxygenated medium
Atto647N STORM	Pyronine dye	5-state	ATTO-TEC	No	Ad-hoc model for Atto647N in STORM buffer
Atto647N ROXS	Pyronine dye	5-state	ATTO-TEC	No	Ad-hoc model for Atto647N in ROXS buffer
Atto655	Oxazine dye	2-state	ATTO-TEC	No	No photophysics
Atto633	Pyronine dye	2-state	ATTO-TEC	No	No photophysics
NileRed	Oxazine dye	2-state	SpectraViewer	No	Fluorogenic
PA-JF549	Photoactivatable Rhodamine	4-state	SMIS based on ref [19]	No	Includes photoactivation and blinking
Bead	-	2-state	SMIS synthetic	No	SMIS created bead

*: All photophysical models include bleaching
$: FPbase: https://www.fpbase.org/
£: ThermoFisher SpectraViewer: https://www.thermofisher.com/order/fluorescence-spectraviewer
%: AAT Spectrum Viewer: https://www.aatbio.com/fluorescence-excitation-emission-spectrum-graph-viewer
#: ATTO-TEC spectra: https://www.atto-tec.com/

Supplementary Figures

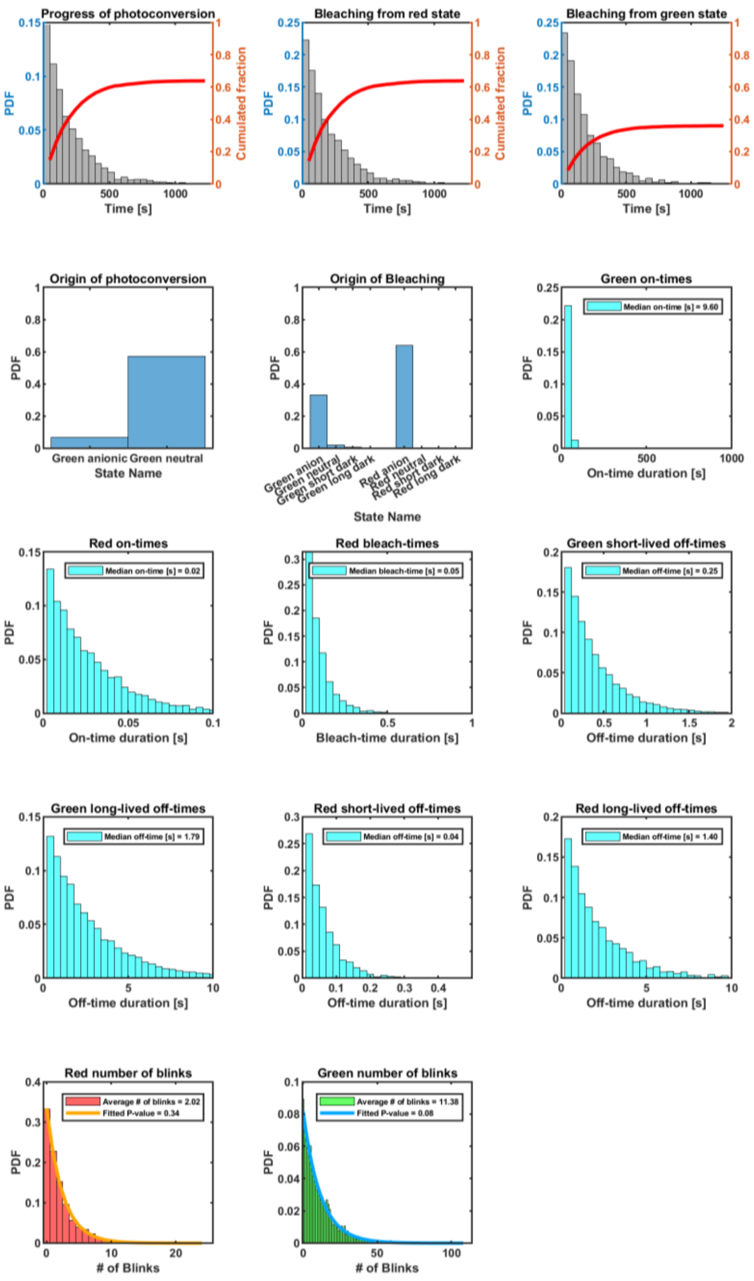

Supplementary Fig. S1

Ground-truth photophysical behavior of mEos4b in SMIS simulations employing the model of Fig. 1a. The case of the simulation in Fig. 1c, Panel 2 is shown (3.5 kW/cm^2, pH 7.5). PDF: probability density function. P-value: probability of bleaching normalized by the probability of transitioning to either bleached or blinked states.

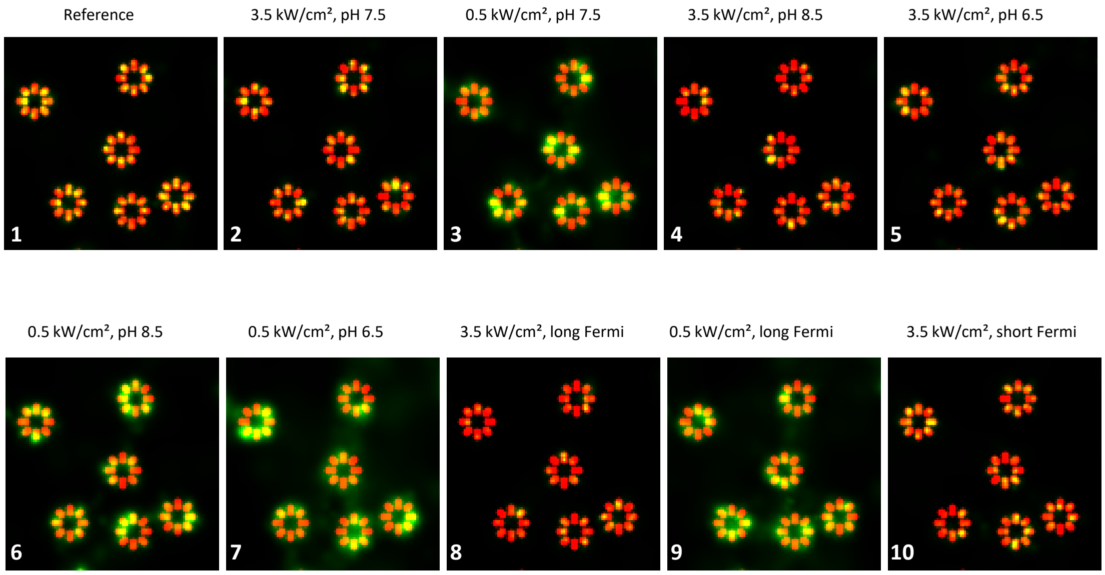

Supplementary Fig. S2

Blend images obtained by combining rendered NPC images shown in Fig. 1c and a ground truth NPC image assuming 100% labeling efficiency and Nup96 Gaussian spots of ~10 nm fullwidth at half maximum (FWHM). The panel numbers shown correspond to those in Fig. 1c. Red color is a marker for incomplete labeling due to lack of mEos4b photoconversion or detection. Green color is a marker for degraded localization precision.

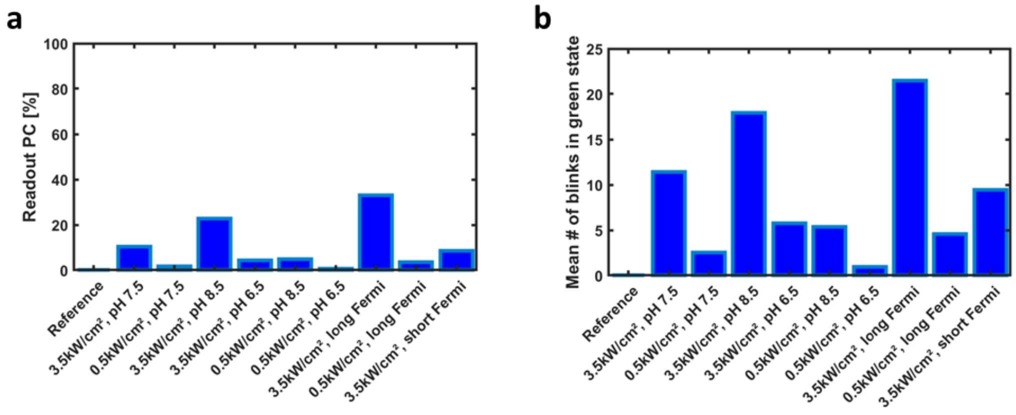

Supplementary Fig. S3

Percentage of mEos4b readout photoconversion (a) and mean number of blinks before photoconversion (b) in the performed SMIS simulations. Values extracted from ground truth SMIS data. Each data point corresponds to n=1 simulation.

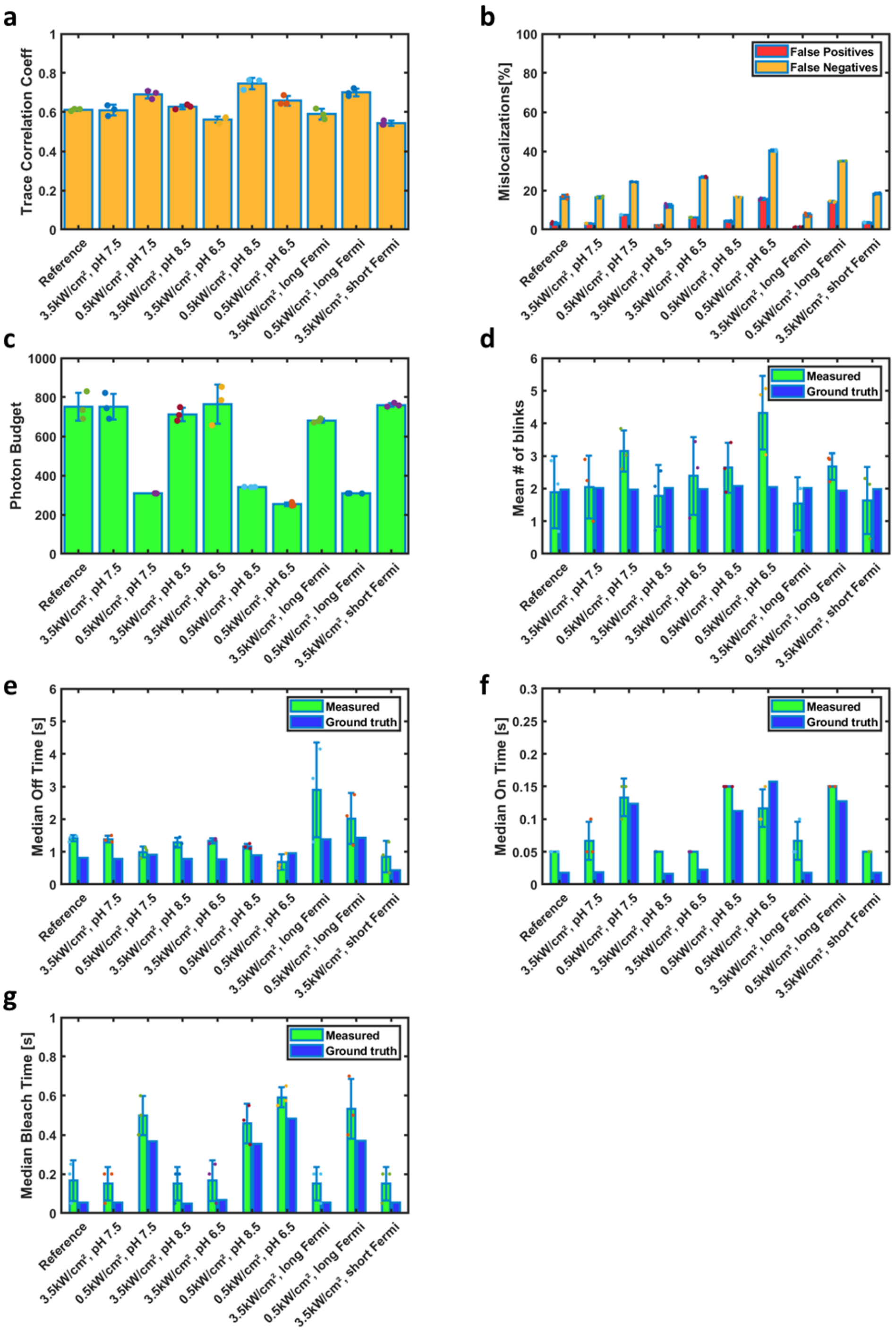

Supplementary Fig. S4

Comparison of ground-truth versus retrieved photophysical data from SMIS simulations of Fig. 1. (a) Median fluorescence-state trace correlation; (b) False-positive (spurious localizations) and false-negative (missed localizations); (c) Median detected photon budget per localization; (d) Mean number of blinks in the photoconverted red state; (e,f,g) Median off-times, on-times and bleach time in the photoconverted red state. Error bars in c-g where computed by splitting the dataset in n=3 subsets along time and calculating standard deviations between the subsets. Colored dots show individual data points.

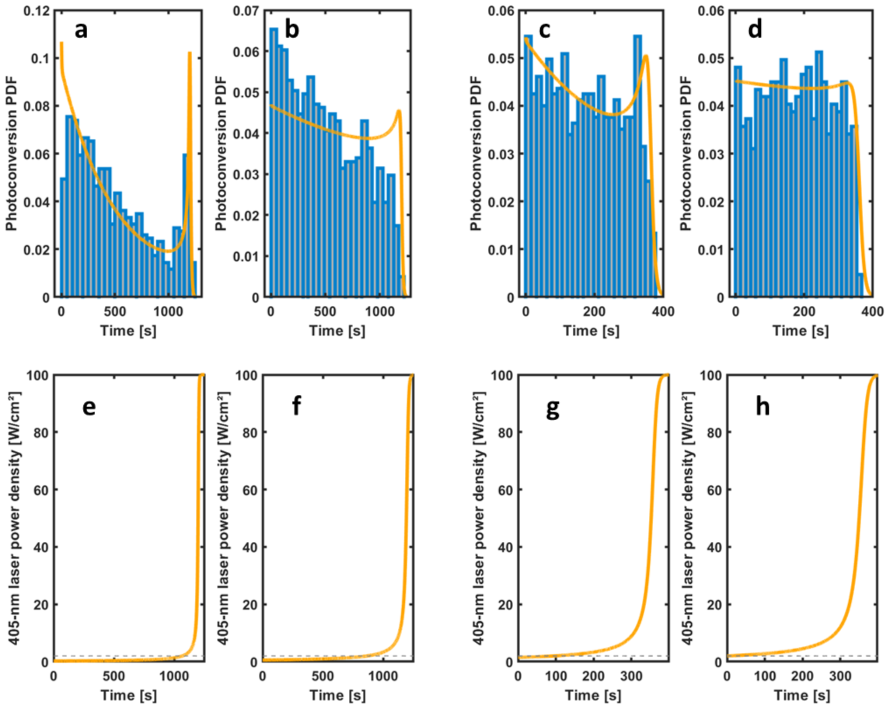

Supplementary Fig. S5

(a-d) Overlay of theoretical Probability Density Function (PDF) of photoconversion (orange) and recovered PDF from processed SMIS data (blue) along data collection schemes using Fermi profiles. (e-h) Corresponding Fermi profiles (orange) employed for the 405-nm laser, by reference to constant laser power density (dashed gray line) used in the other NPC SMIS simulations described in this work. (a,e) 3.5 kW/cm², long Fermi (1200 s), (b,f) 0.5 kW/cm², long Fermi (1200 s), (c,g) 3.5 kW/cm², short Fermi (400 s), (d,h) 0.5 kW/cm², short Fermi (400 s)

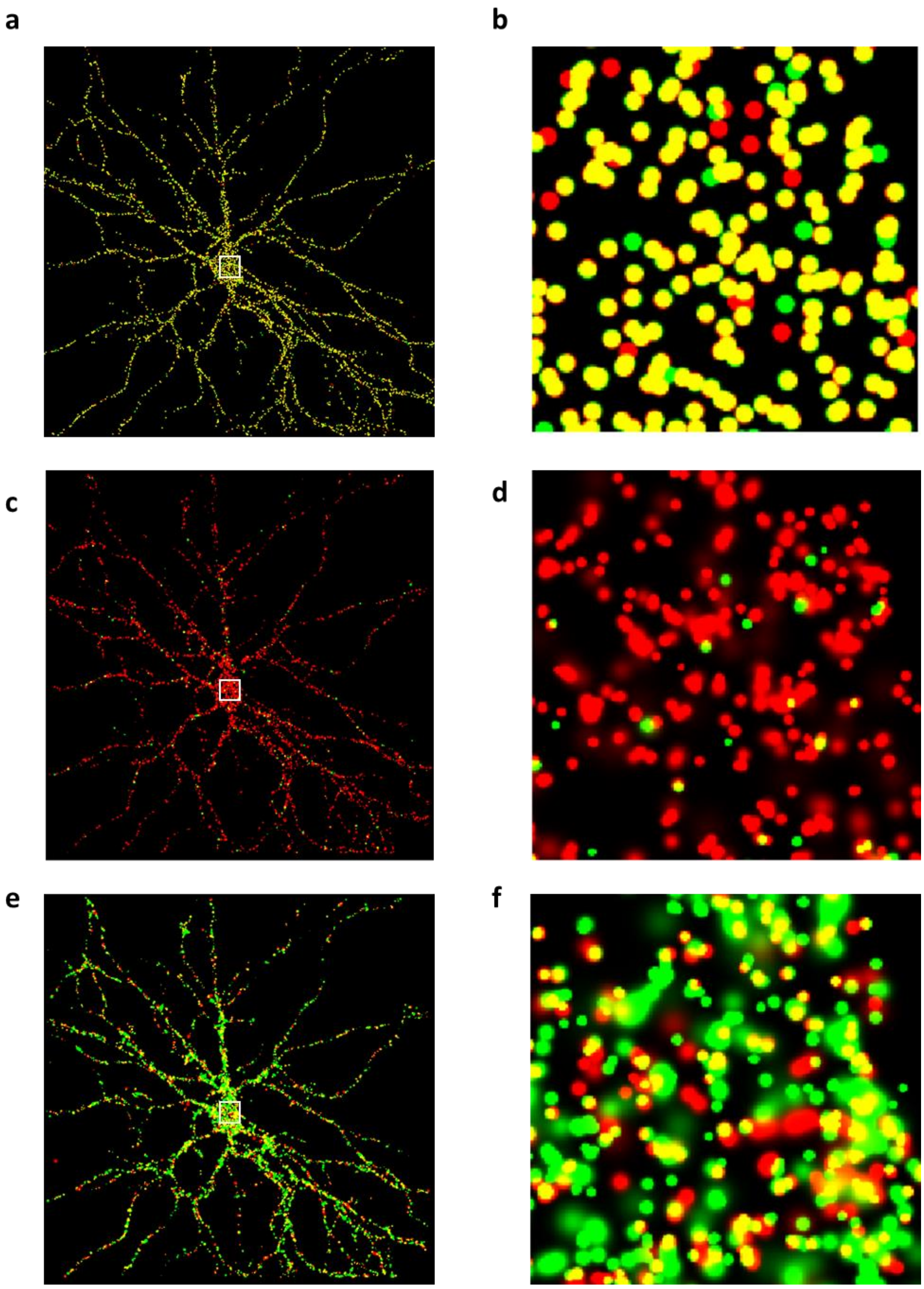

Supplementary Fig. S6

Pseudo-2 color SMIS simulations related to Fig. 2. (a-b) Ground truth colocalization of mEos4b (green) and Dendra2 (red) (c-d) Colocalization of mEos4b (green, in absence of mEos4b green-state photoswitching) and Dendra2 (red), (e-f) Colocalization of PAmCherry (green) and Dendra2 (red). Areas within the white squares in (a,c,e) are enlarged in (b,d,f) for inspection of colocalization events.

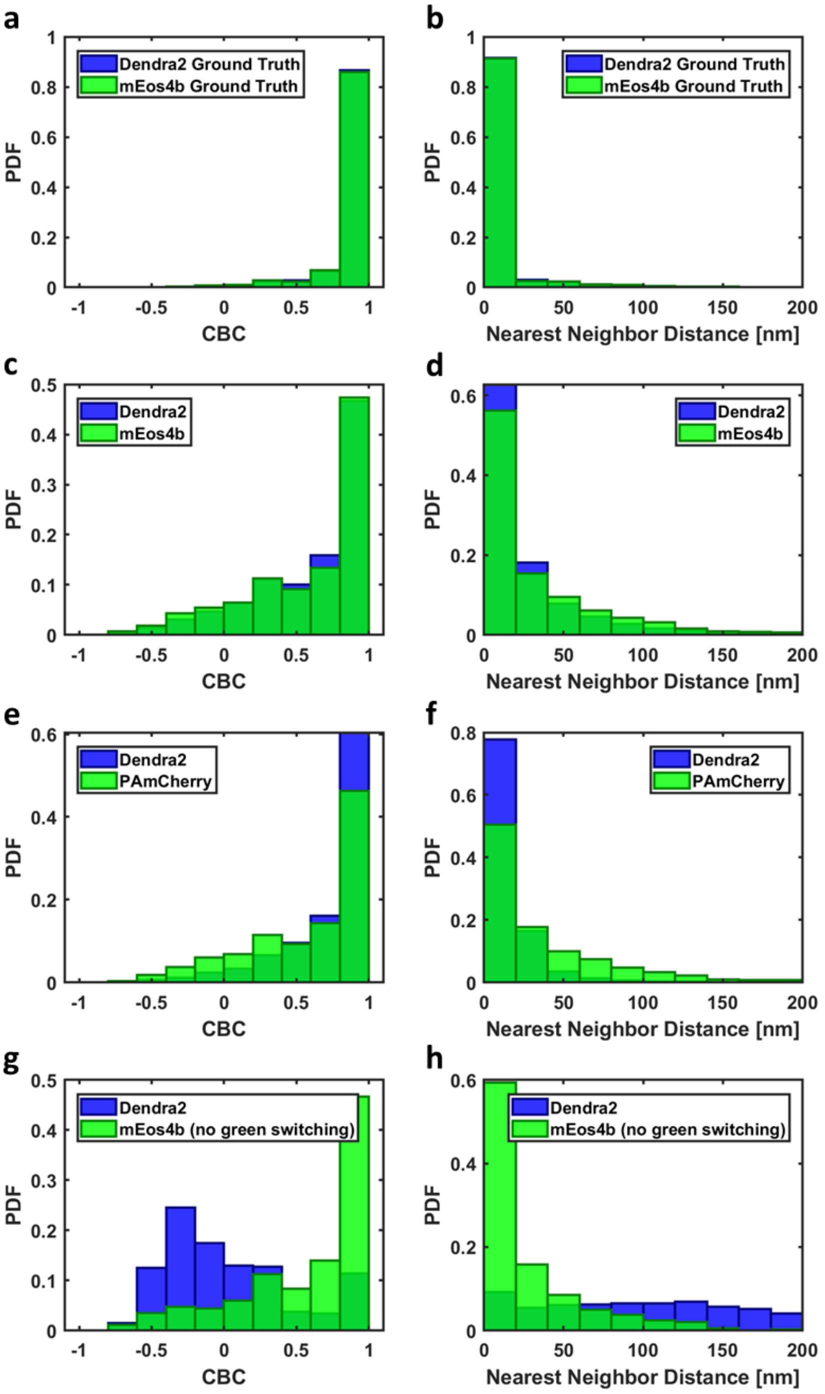

Supplementary Fig. S7

Histograms of coordinate based colocalizations (CBC) indices and nearest neighbor distances for the various fluorescent protein pairs tested. (a-b) Ground truth colocalization of mEos4b and Dendra2. (c-d) Measured colocalization of mEos4b and Dendra2. (e-f) Measured colocalization of PAmCherry and Dendra2. (g-h) Measured colocalization of mEos4b (no green state photoswitching) and Dendra2. PDF : probability density function.

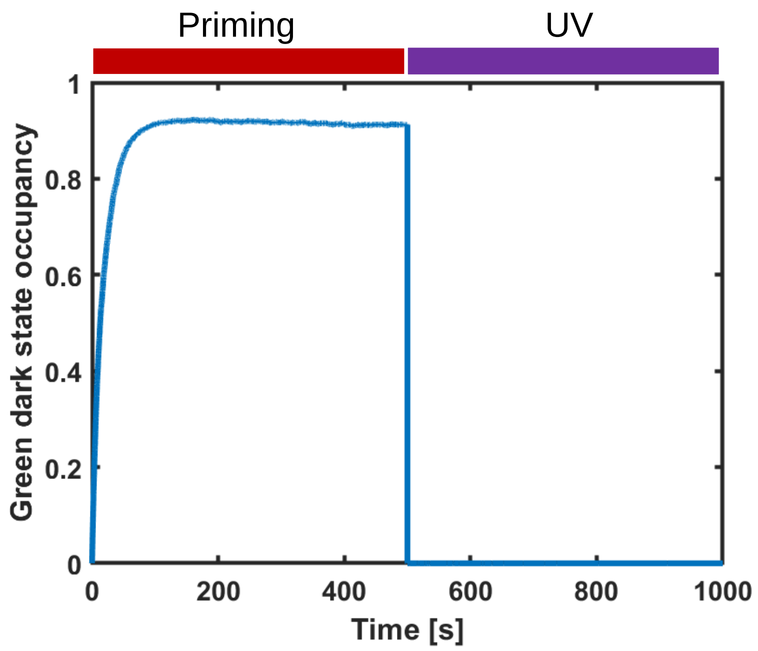

Supplementary Fig. S8

Occupancy of the mEos4b long-lived dark state during pseudo-2 color SMIS data collection (priming phase followed by UV phase).

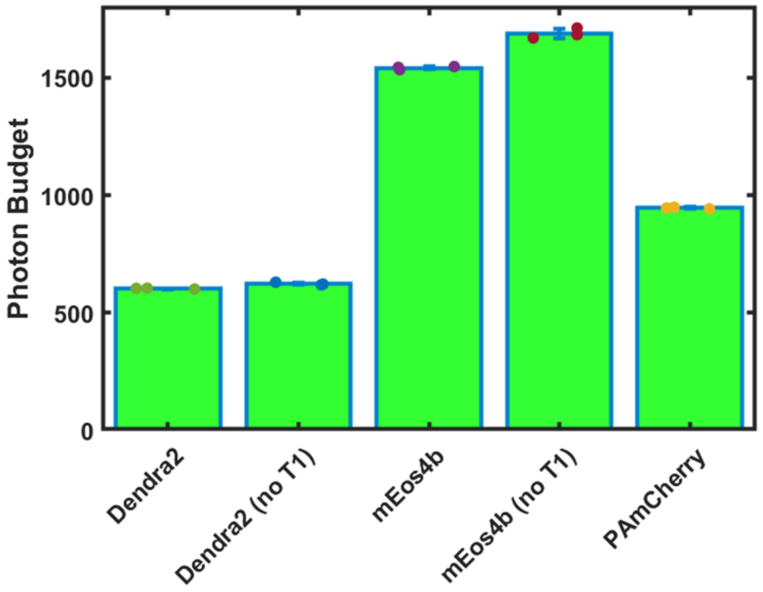

Supplementary Fig. S9

Photon-budget per localization during pseudo-2 color SMIS data collection. In Dendra2 (no T1) and mEos4b (no T1), intersystem crossing of the red chromophore to the triplet state was artificially suppressed, resulting in higher photon budgets per localization.

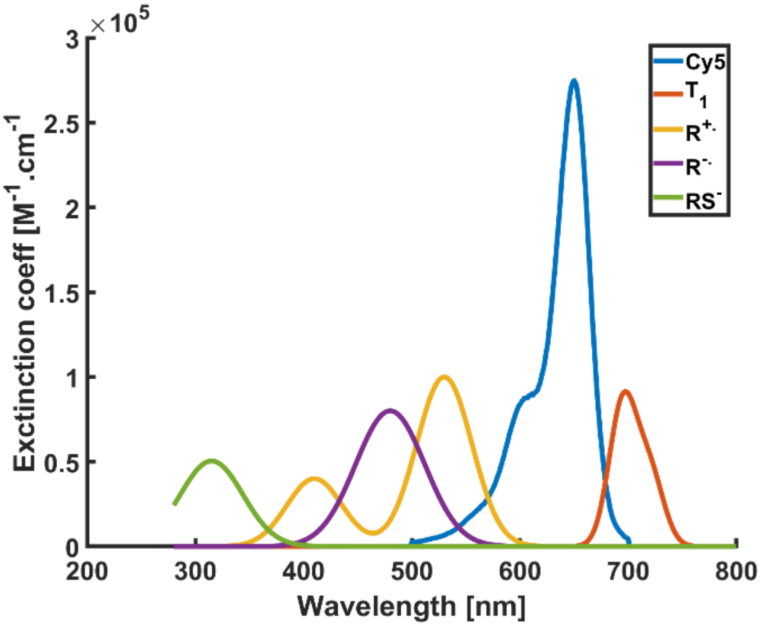

Supplementary Fig. S10

Absorption spectra of Cy5 photophysical states used in dSTORM SMIS simulations of Fig. 3. Spectra were generated with the dedicated SMIS tool, based on spectral data of Fig. 3 in Gidi *et al*.[3]

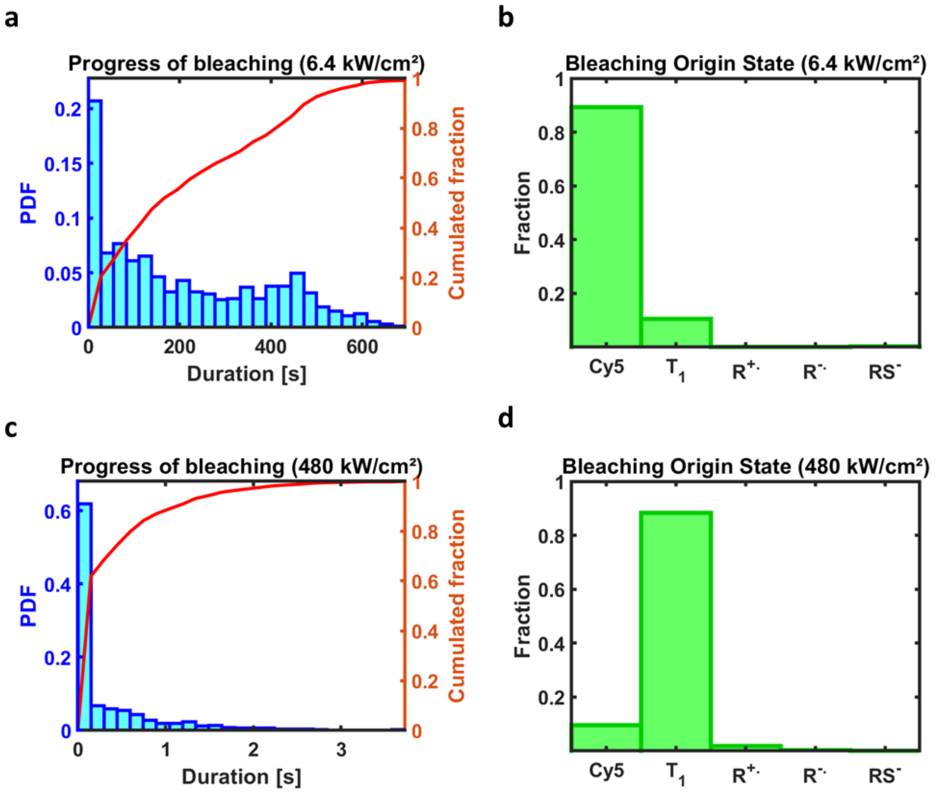

Supplementary Fig. S11

Progress of Cy5 photobleaching (a,c) and photophysical state at the origin of bleaching (b,d) with 6.4 kW/cm^2 (a,b) or 480 kW/cm^2 (c,d) 647 nm laser power density. PDF : probability density function. Ground truth data are shown for n=1 simulation.

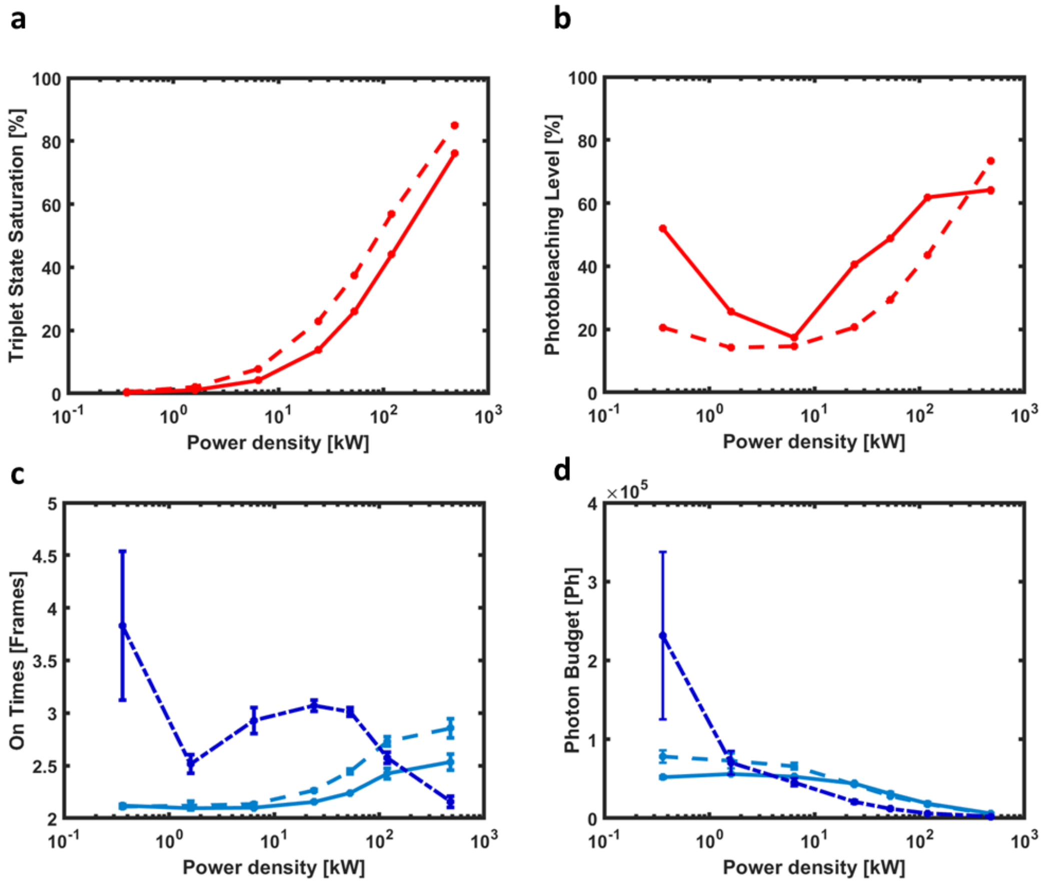

Supplementary Fig. S12

Triplet-state saturation level of Cy5 (a) and photobleaching level reached after illumination for 1000 frames (b), as a function of 647 nm light power density, using photophysical parameters of Gidi *et al.*[3] (plain lines) or corrected parameters (dashed line). On times (c) and photon budget (d) from experimental data from Diekmann *et al.*[6] (dark blue dashed line), or from SMIS simulations using photophysical parameters of Gidi *et al.*[3] (light blue plain lines) or corrected parameters (light blue dashed line). (a,b) Ground truth data are shown for n=1 simulation. (c,d) Error bars for SMIS data show standard deviation for n=3 simulations. Individual data points not shown for clarity.

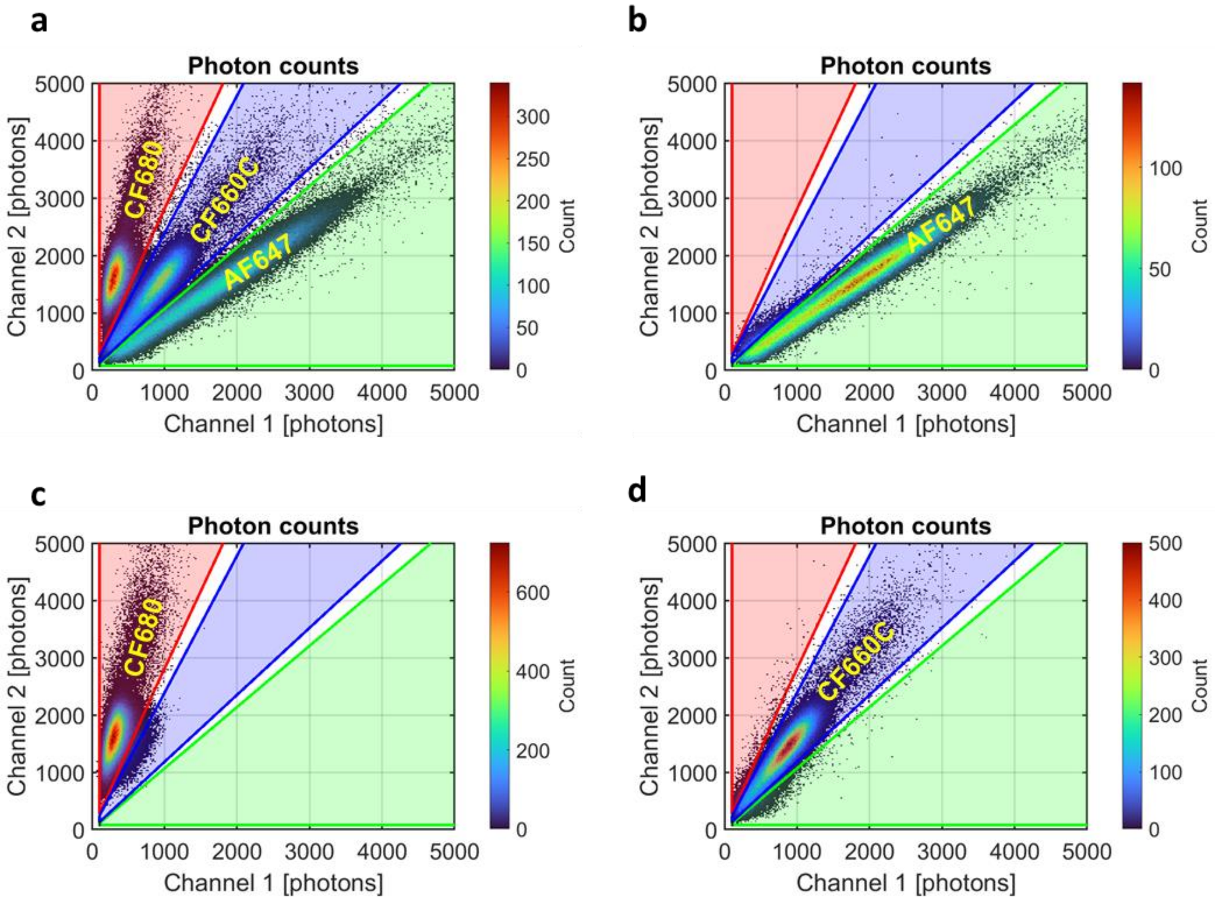

Supplementary Fig. S13

Bivariate histograms of photon counts for spectral demixing of AF647, CF660C and CF680. The numbers of fluorophores with specified numbers of detected photons in the two channels (channel 1: shorter wavelengths, channel 2: longer wavelengths) are shown according to color maps displayed on the right of the plots. Regions of interest (ROIs) for unmixing are shown in red, green and blue. (a) 3-color dataset. (b) control for AF647. (c) control for CF680. (d) control for CF660C.

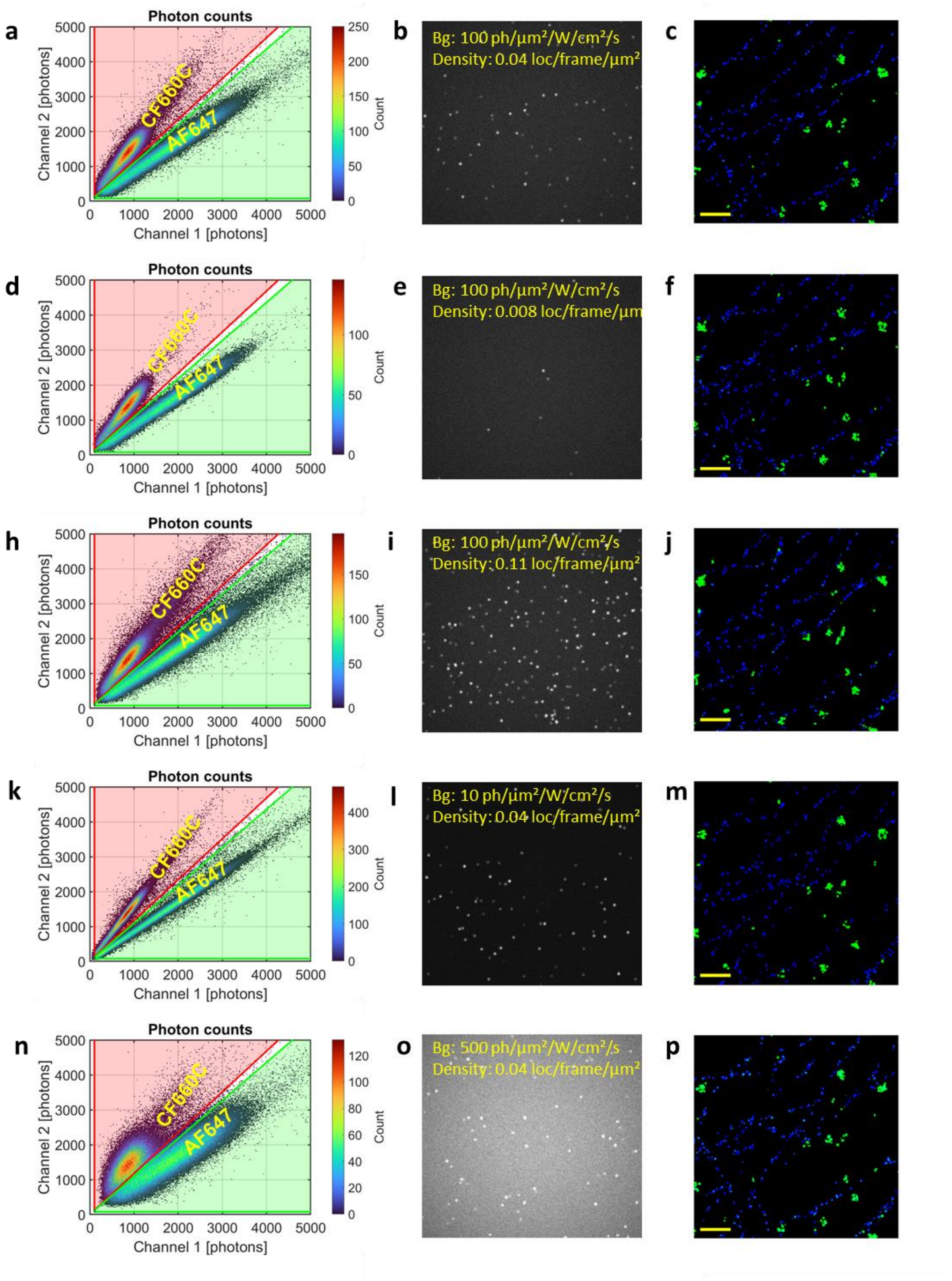

Supplementary Fig. S14

Sensitivity of spectral demixing to localization density and fluorescence background in 2-color experiments with AF647 and CF660C. From top to bottom: medium background/ medium localization density; medium background/ low localization density; medium background/ high localization density; low background/ medium localization density; high background/ medium localization density. (a,d,h,k,n): Bivariate histograms of photon

counts. The numbers of fluorophores with specified numbers of detected photons in the two channels (channel 1: shorter wavelengths, channel 2: longer wavelengths) are shown according to color maps displayed on the right of the plots. Regions of interest (ROIs) for unmixing are shown in red and green. (b,e,i,l,o) raw SMIS frame (frame #100) acquired in channel 1 for each condition indicated in yellow. Bg: background fluorescence, Density: localization density. (c,f,j,m,p) extracts of rendered 2-color images for each condition. Blue: AF647; Green: CF660C. Scale bar: 1 μm.

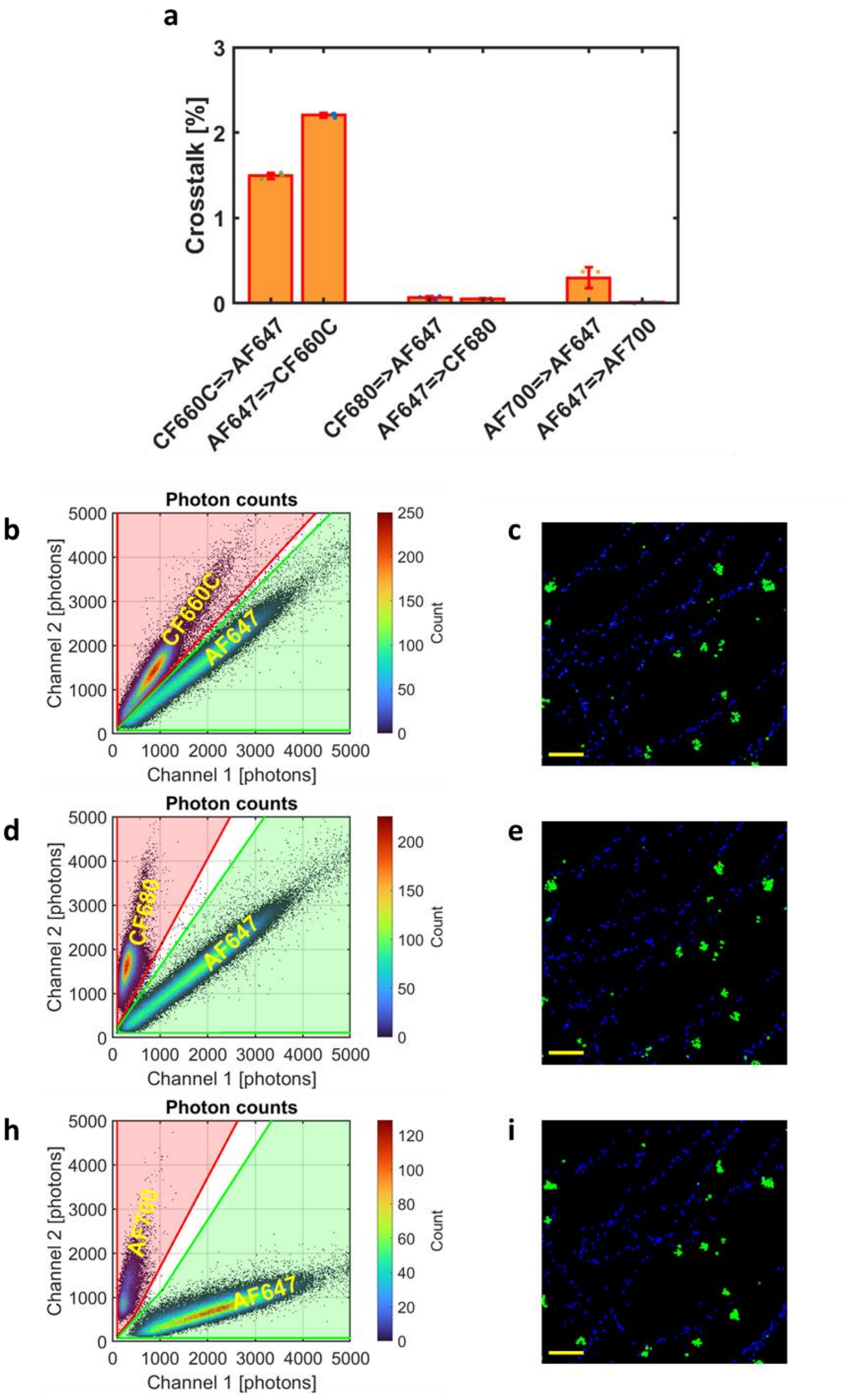

Supplementary Fig. S15

Sensitivity of spectral demixing to fluorophore photophysics in 2-color experiments with AF647 in tandem with CF660C, CF680 or AF700. (a) Crosstalk between pairs of fluorophores. Error bars were computed by randomly splitting simulated datasets in n=3 subsets. Colored dots show individual data points. (b,d,h): Bivariate histograms of photon counts. The numbers of fluorophores with specified numbers of detected photons in the

two channels (channel 1: shorter wavelengths, channel 2: longer wavelengths) are shown according to color maps displayed on the right of the plots. Regions of interest (ROIs) for unmixing are shown in red and green. (c,e,i) extracts of rendered 2-color images. Blue: AF647; Green: CF660C (c), CF680 (e), AF700 (i). Scale bar: 1 μm.

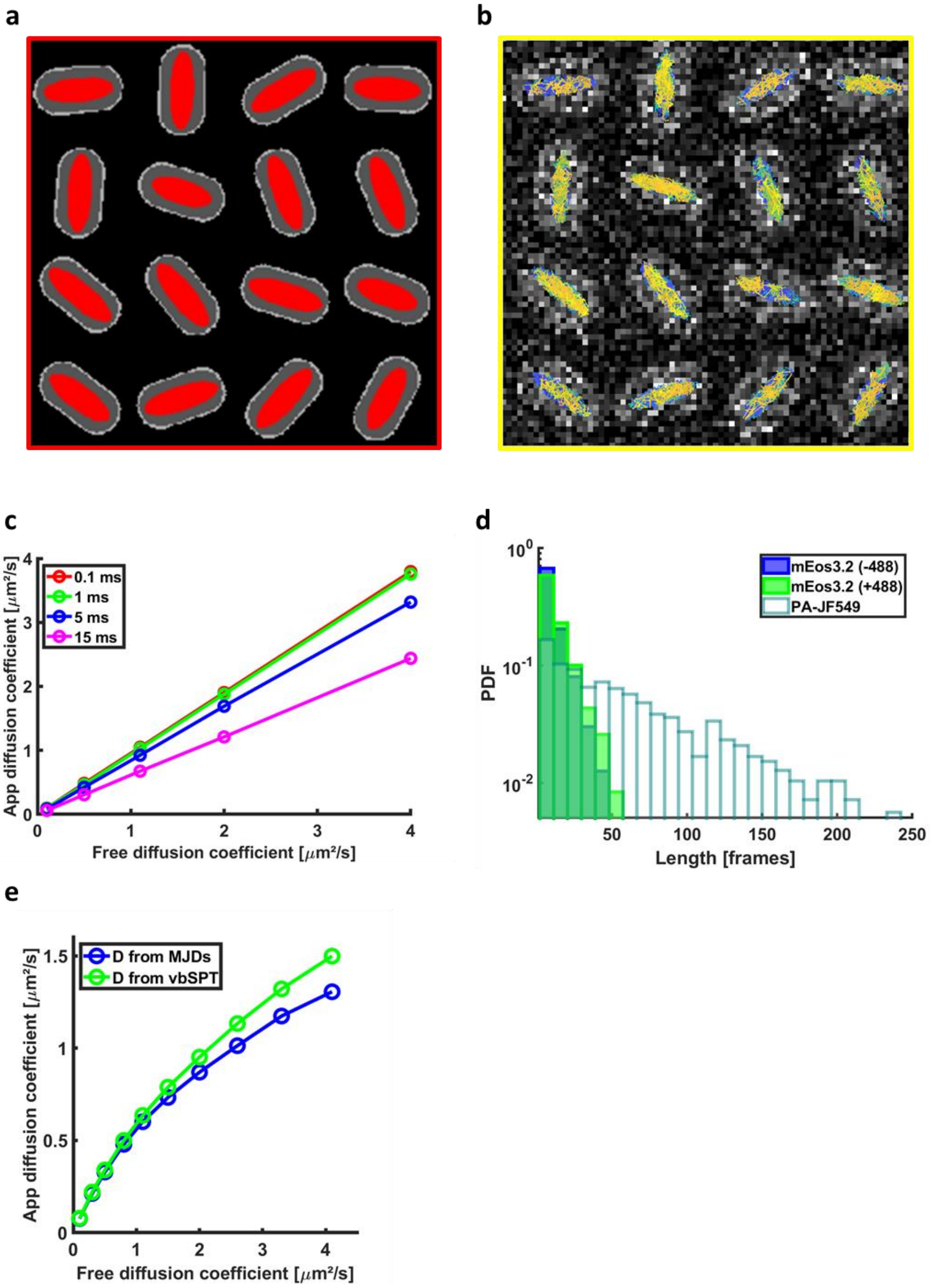

Supplementary Fig. S16

(a) Extract of the bacterial field used for the sptPALM simulations of Fig. 5, highlighting in red the nucleoid in which the labeled NAP diffuse. (b) Same area of the bacterial field virtual sample highlighting mEos3.2 tracks superimposed on a SMIS-generated CCD frame showing autofluorescence produced by individual bacteria. Figure produced with Trackit[20]. (c) Effect of temporal averaging on non-confined diffusing mEos3.2 molecules. Input free diffusion coefficients are shown on the X-axis, whereas apparent diffusion coefficients extracted from

MJD histograms are shown on the Y-axis. Apparent diffusion coefficients are smaller when progressively increasing CCD exposure while progressively decreasing dark times between frames, at constant total frame time (15 ms). (d) Tracklength histograms of mEos3.2 in the absence or presence of additional 488 nm light, compared to PA-JF549 measured upon processing of the generated SMIS image stacks. Each histogram from n=1 simulation. (e) Calibration curves assuming single populations of fluorophores moving with increasing diffusion coefficients within the confined *E. coli* nucleoid, and showing measured apparent diffusion coefficients versus input free diffusion coefficients, at fixed frame time (5 ms CCD exposure + 10 ms dark time). PDF: probability density function. (c,e) Each data point from n=1 simulation.

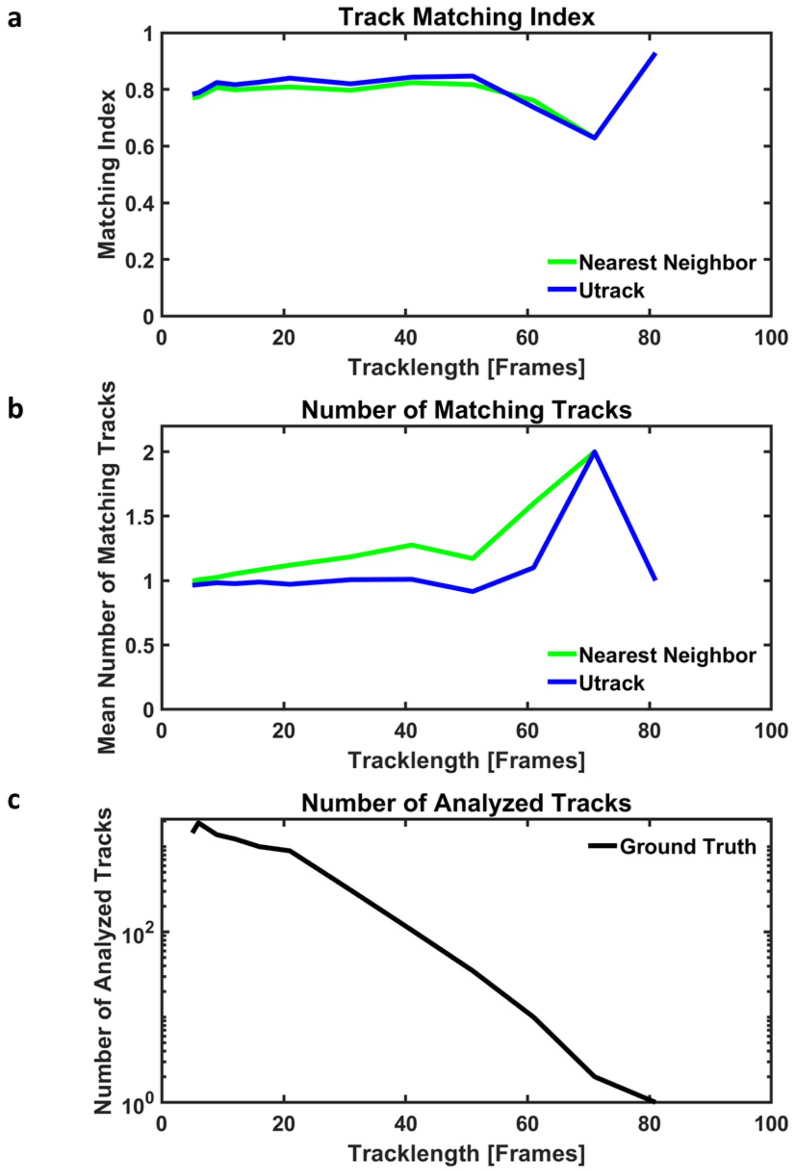

Supplementary Fig. S17

Example of the quality of track retrieval by Trackit[20], using a nucleoid-confined mEos3.2 SPT SMIS dataset (n=1) with 15 ms total frame time, and comparison between Nearest Neighbor (green) and UTrack (blue). (a) Track matching index: For each ground truth track (allowing a blinking gap of 2 frames), the closest experimental matching track is considered and the fraction of the ground truth localizations for which a processed localization is found at a distance < 30 nm is computed. (b) Number of matching tracks: for each ground truth track, the number of matching experimental tracks (i.e. for which a localization is found at a distance < 30 nm from a ground truth localization) is computed. (c) Number of ground truth analyzed tracks.

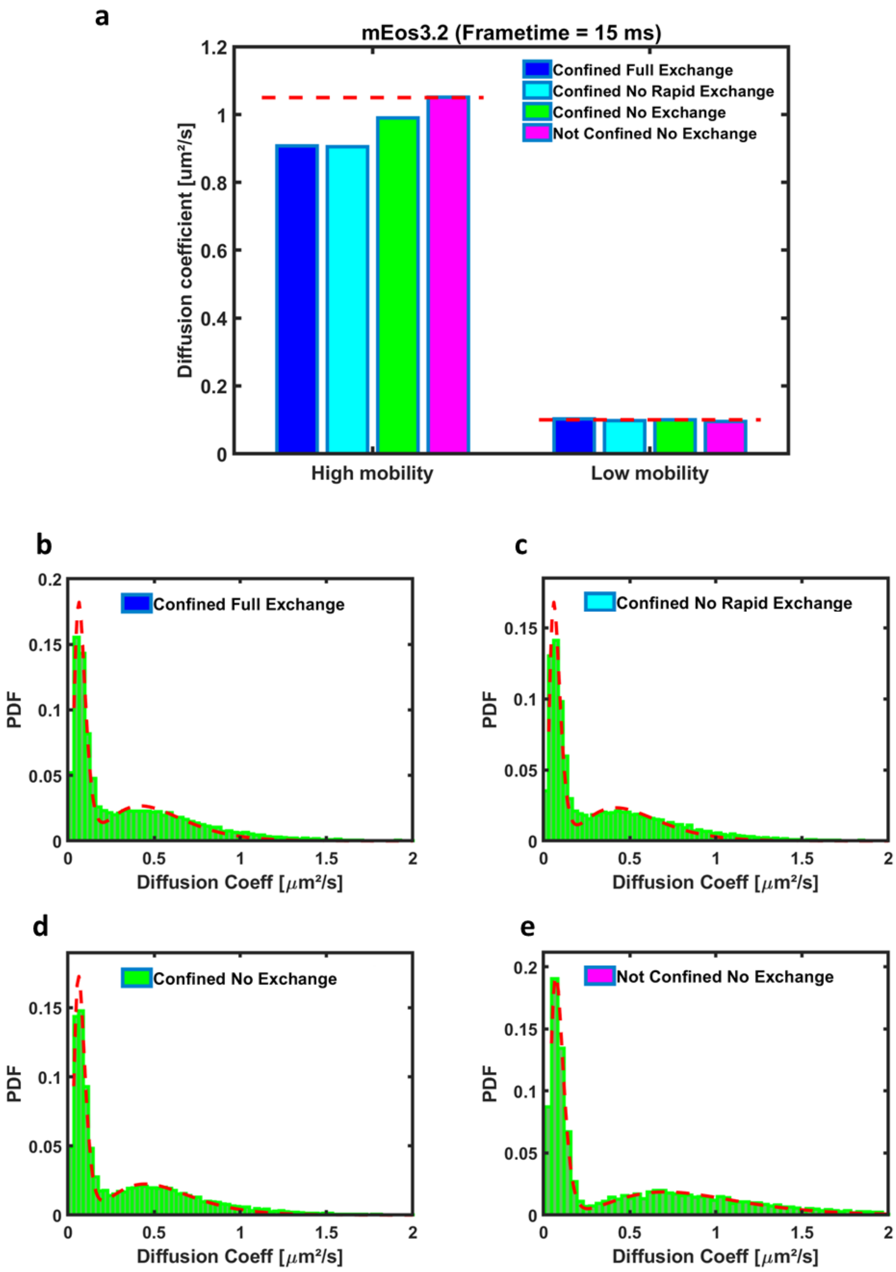

Supplementary Fig. S18

Effect of kinetic exchange and confinement on retrieved unbiased diffusion coefficients from mEos3.2 SPT data (in the absence of 488 nm light) extracted from MJD-based diffusion coefficient histograms (tracklength = 5, total frametime = 15 ms). (a) Retrieved unbiased diffusion coefficients for the low and high mobility populations. Ground truth values are marked as dashed red lines. Nucleoid confined mEos3.2 with both slow and rapid exchange as in Fig. 5a (blue); Nucleoid confined mEos3.2 with only slow exchange (cyan); Nucleoid confined mEos3.2 without exchange (green); Non-confined mEos3.2 without exchange (magenta). Each data point from n=1 simulation. Associated apparent diffusion coefficient histograms (green) are shown in (b-e), together with associated fits from a 2-state model (red). The quality of the fits progressively improves from panel b to panel e.

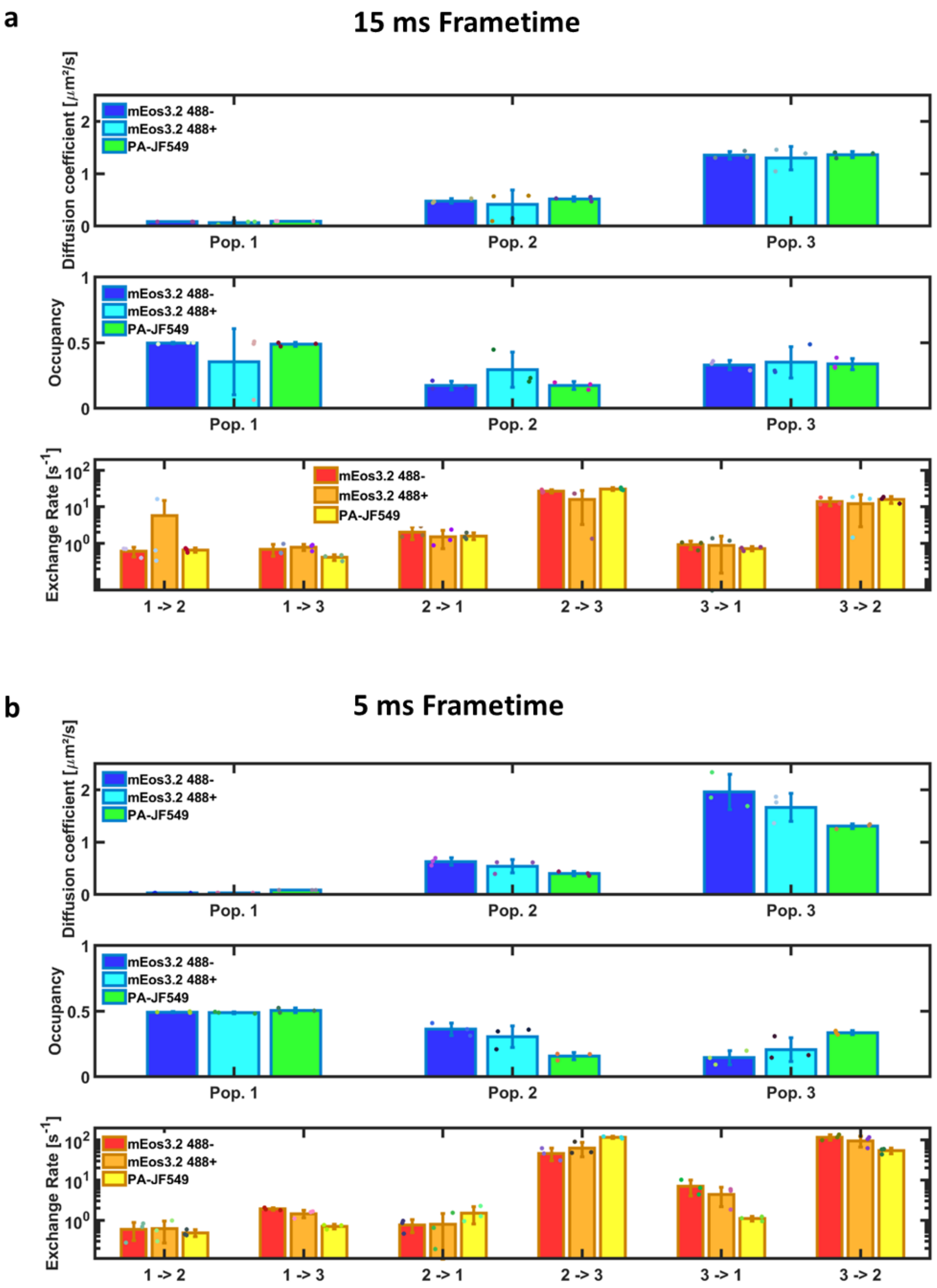

Supplementary Fig. S19

Retrieved unbiased diffusion coefficients, population occupancies and exchange rates output by vbSPT as the most likely diffusion model. (a) 15 ms total frametime. (b) 5 ms total frametime. In both cases, output values significantly deviate from ground truth values, show substantial standard deviations, and differ substantially between (a) and (b), suggesting that the 3-state models are false. Error bars show standard deviation for n=3 simulations. Colored dots show individual data points.

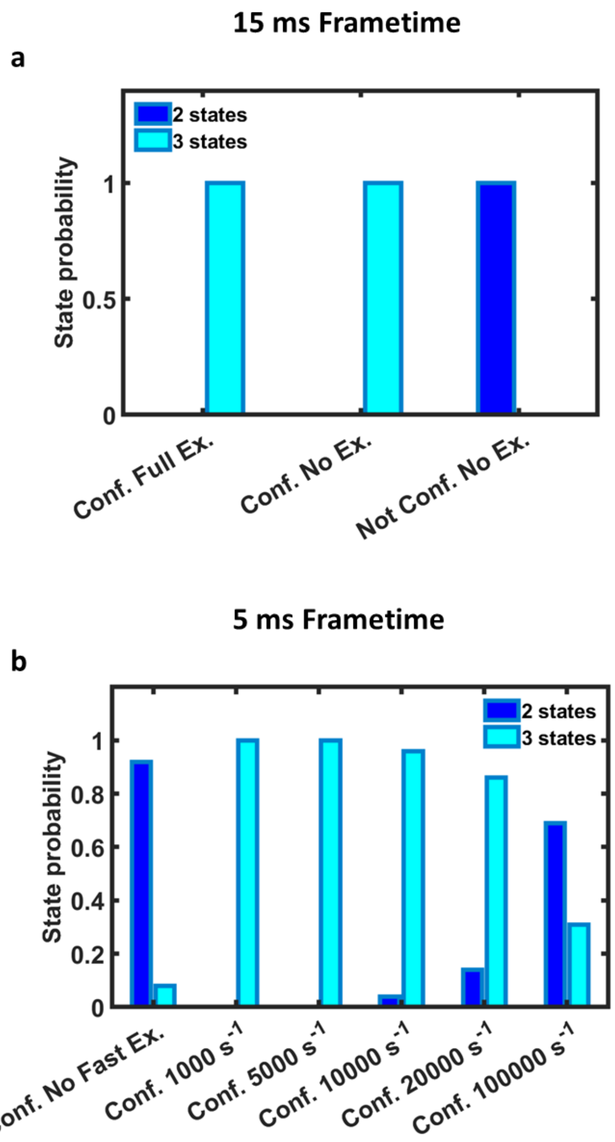

Supplementary Fig. S20

Probability of 2-state (dark blue) versus 3-state (light blue) models assessed by bootstrapping in vbSPT. (a) mEos3.2 SPT SMIS datasets in the absence of 488-nm light, 15 ms total frametime. Nucleoid confined mEos3.2 with both slow and rapid exchange as in Fig. 5a (Conf. Full Ex.); Nucleoid confined mEos3.2 without exchange (Conf. No Ex.); Non-confined mEos3.2 without exchange (Not Conf. No Ex.). At 15 ms total frametime, nucleoid confinement is sufficient to produce a false 3-state model. (b) PA-JF549 SPT SMIS datasets, 5 ms total frametime. The case of nucleoid confined PA-JF549 only slow exchange (Conf. No Fast Ex.) is compared to cases with both slow and rapid exchange from 1000 s^{-1} to 100000 s^{-1}. A 2-state model is favored only in the absence of rapid exchange, or in the presence of very fast exchange. Each data point from n=1 simulation.

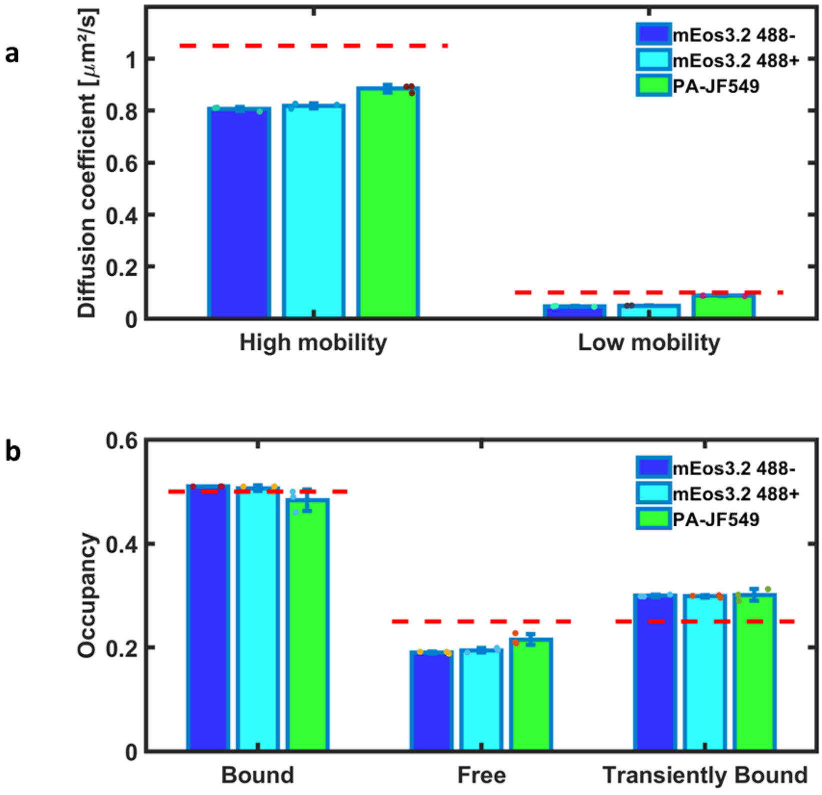

Supplementary Fig. S21

Unbiased diffusion coefficients (a) and diffusion state's occupancies (b) recovered from MJD's-derived diffusion coefficients for mEos3.2 in the absence (blue) or presence (cyan) of 488 nm light, and for PA-JF549 (green). Ground truth values are shown as dashed red lines. Total frame time = 5 ms, to be compared with Fig. 4D. Error bars show standard deviation for n=3 simulations. Colored dots show individual data points.

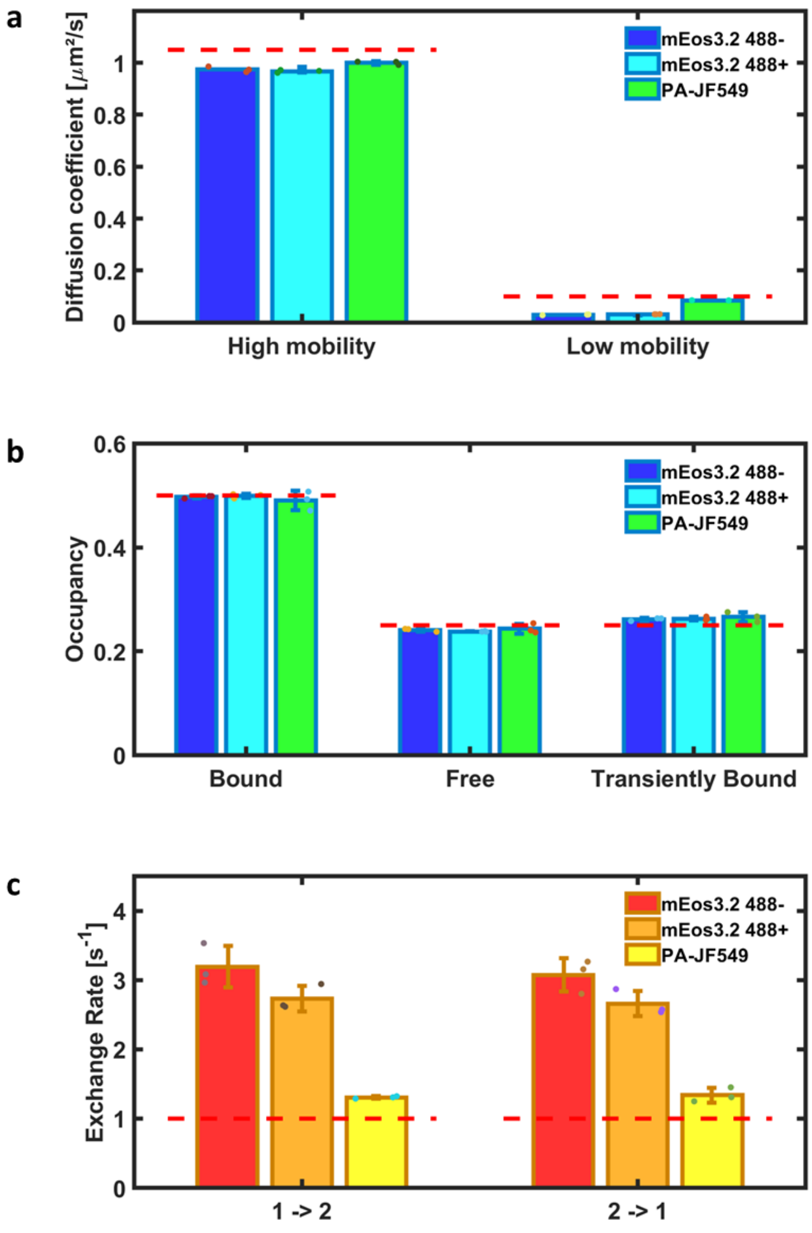

Supplementary Fig. S22

Unbiased diffusion coefficients (a) and diffusion state's occupancies (b) recovered from vbSPT-derived diffusion coefficients for mEos3.2 in the absence (blue) or presence (cyan) of 488 nm light, and for PA-JF549 (green). (c) Slow exchange rates recovered from vbSPT, for mEos3.2 in the absence (red) or presence (orange) of 488 nm light, and for PA-JF549 (yellow). 1 $\rightarrow$ 2: Tight DNA-bound state to searching state; 2 $\rightarrow$ 1: Searching state to tight DNA-bound state. Total frame time = 5 ms, to be compared with Fig. 5e-f. Error bars show standard deviation for n=3 simulations. Colored dots show individual data points.

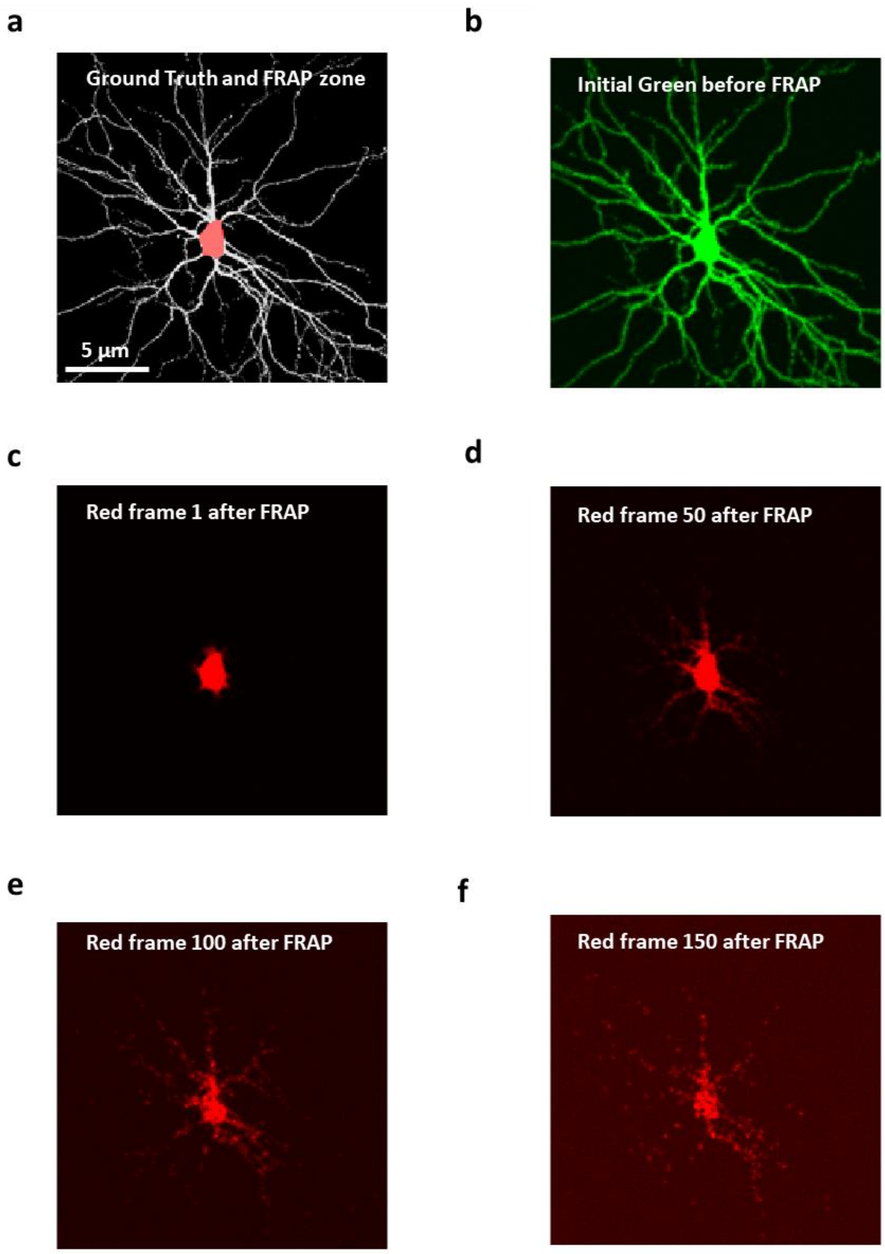

Supplementary Fig. S23

SMIS pulse-chase experiment with mEos4b. (a) Virtual 2D neuronal cell (grey) and photoconversion zone (pink) applied at the level of the cell nucleus. (b) Initial mEos4b green image before FRAP (green channel of the virtual microscope). (c-f) mEos4b red images (red channel of the virtual microscope) immediately after photoconversion in the FRAP zone (c) and 2.5 s (d), 5 s (e) and 7.5 s (f) after photoconversion. Diffusion of the photoconverted mEos4b red molecules is clearly visible, together with progressive photobleaching. Frametime = 50 ms ; mEos4b diffusion coefficient : 2 $\mu m^2/s$. 561-nm laser power density : 200 W/cm^2 ; 488-nm laser power density : 100 W/cm^2 (1st frame) ; 405-nm laser power density: 5000 W/cm^2 (2nd frame, FRAP zone).

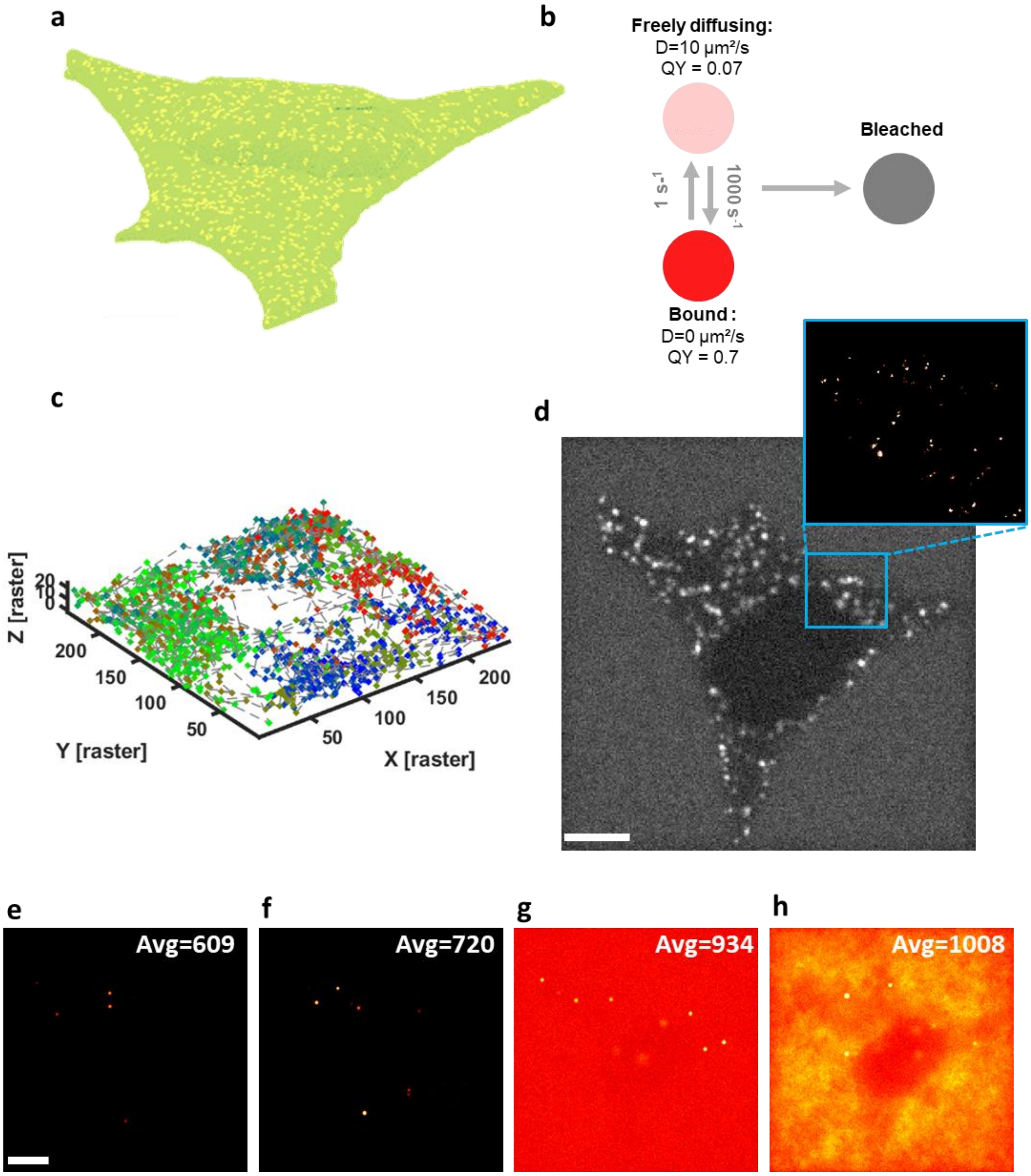

Supplementary Fig. S24

SMIS 3D-PAINT imaging of receptor clusters anchored in the cytoplasmic membrane of a HeLa cell, using fluorogenic NileRed and HILO illumination mode. (a) 3D view of the virtual HeLa cell. Receptor clusters are drawn in yellow. (b) Employed NileRed photophysical and diffusion model (QY = fluorescence quantum yield). (c) Exemple of a single NileRed molecule diffusing around the cell for ~1800 frames (from blue to red). (d) Diffraction limited average image of the 10000 recorded frames and view of PAINT reconstructed receptor clusters (zoom from blue square in c). Scalebar = 5 µm. (e-h): Average images of 100-frames PAINT datasets. Average image counts are shown. Images are shown on the same scale. Scalebar = 5 µm. (e) Hilo mode, fluorogenic labels; (f) Hilo mode, non-fluorogenic labels. Fluorescence from the non-bound labels rapidly diffusing in the HILO zone

slightly increases the average background fluorescence; (g) Widefield mode, fluorogenic labels; (h) Widefield mode, non-fluorogenic labels. Fluorescence from the non-bound labels rapidly diffusing in 3D is clearly visible and further increases the average background fluorescence. Frametime = 50 ms. 561-nm laser power density : 200 W/cm^2. Autofluorescence : 300 ph/pixel/s.

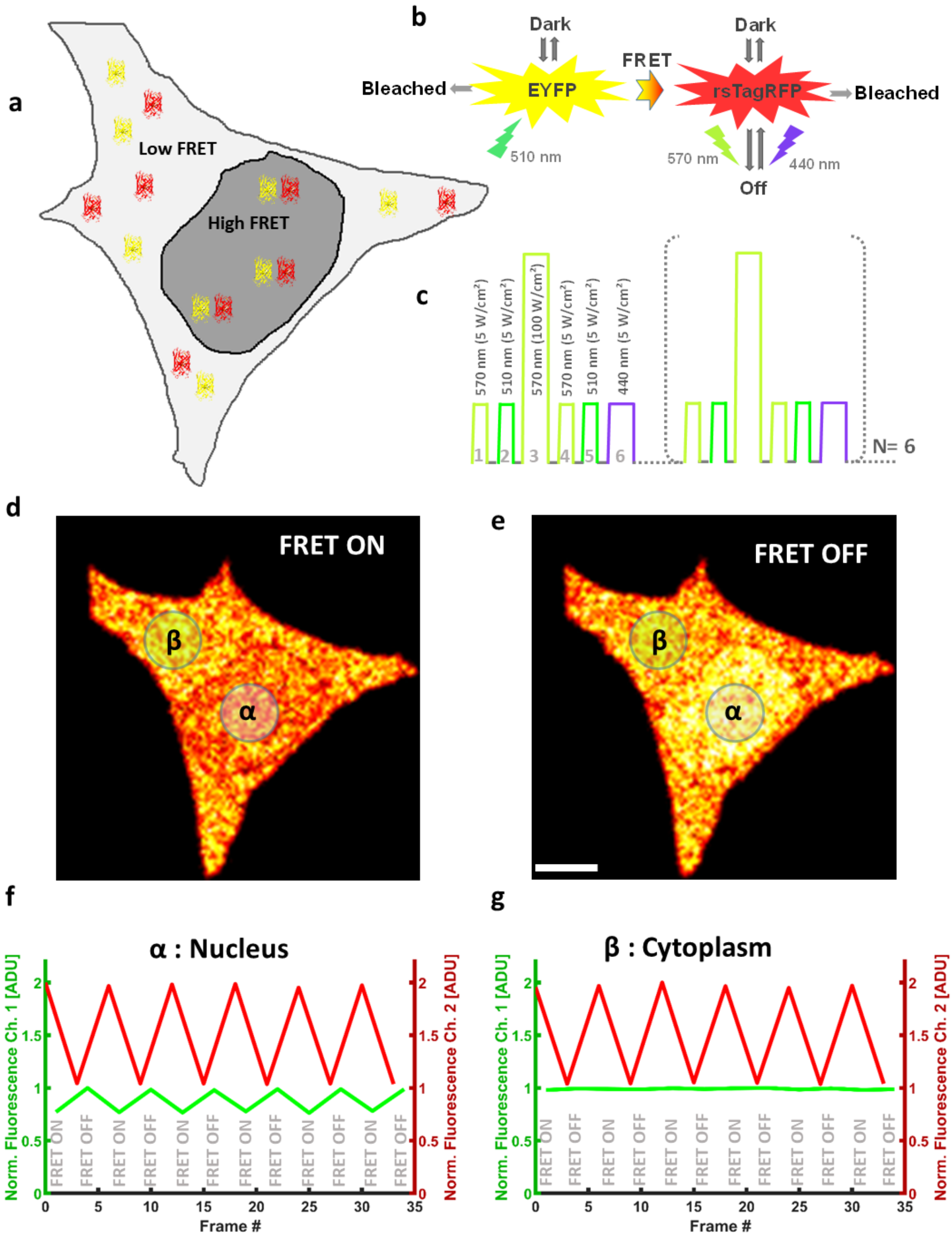

Supplementary Fig. S25

SMIS photochromic FRET experiment with EYFP and rsTagRFP. (a) 2D view of the virtual HeLa cell. EYFP and rsTagRFP colocalize in the nucleus (high FRET, distance 6 nm +/- 1 nm), but not in the cytoplasm (low FRET). (b) Employed photophysical model. Both EYFP and rsTagRFP can bleach or transiently enter a short-lived dark state that thermally recovers at 20 s^{-1}. rsTagRFP photoswitches under 570 nm (on to off) or 440 nm (off to on) light illumination, respectively. (c) Used laser pulse sequence : pulses 1 and 4 (570 nm) correspond to readout

frames to monitor the state of rsTagRFP. Pulses 2 and 5 (510 nm) correspond to readout frames to monitor the state of EYFP. Pulse 3 (570 nm) and 6 (440 nm) are used to switch rsTagRFP off and on, respectively. (d-e) : Fluorescence image recorded in the EYFP channel from pulse 2 ((d), rsTagRFP mostly in the on-state) and 5 ((e), rsTagRFP mostly in the off-state). Regions α and β delineate regions of interest from which the average recorded fluorescence intensity is plotted in f and g. (f-g) Evolution of the EYFP normalized fluorescence (green, left axis, EYFP channel) from region α (f) and β (g) measured by pulses 2 (high FRET) and 5 (low FRET). The lower signal with pulses 2 is a manifestation of a FRET efficiency of ~ 20%. The evolution of the rsTagRFP normalized fluorescence (red, right axis, rsTagRFP channel, offset of 1 unit added) shows photoswitching recorded by pulses 1 (on-switched) and 4 (off-switched). Frametime = 50 ms.

a

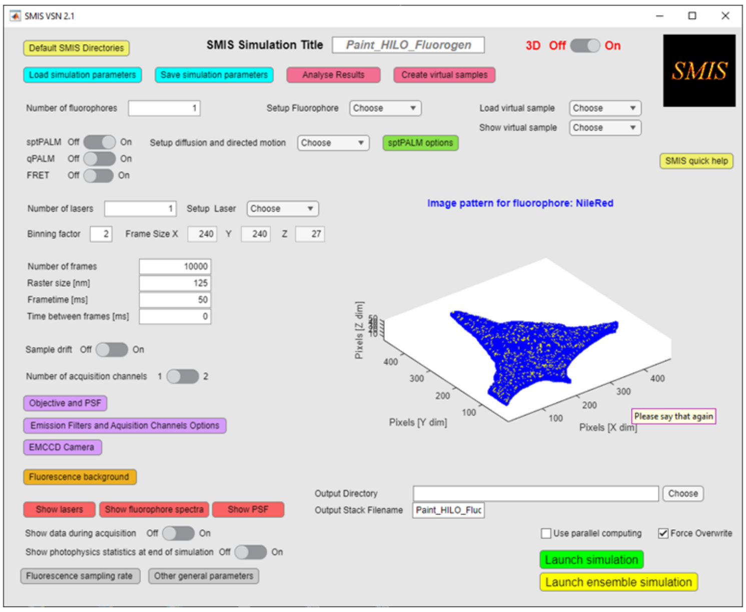
SMIS VSN 2.1
Default SMIS Directories
SMIS Simulation Title
Paint_HILO_Fluorogen
3D Off On
SMIS
Load simulation parameters
Save simulation parameters
Analyse Results
Create virtual samples
Number of fluorophores 1
Setup Fluorophore Choose
Load virtual sample Choose
Show virtual sample Choose
sptPALM Off On
qPALM Off On
FRET Off On
Setup diffusion and directed motion Choose
sptPALM options
SMIS quick help
Number of lasers 1
Setup Laser Choose
Image pattern for fluorophore: NileRed
Binning factor 2
Frame Size X 240 Y 240 Z 27
Number of frames 10000
Raster size [nm] 125
Frametime [ms] 50
Time between frames [ms] 0
Sample drift Off On
Number of acquisition channels 1 2
Objective and PSF
Emission Filters and Aquisition Channels Options
EMCCD Camera
Fluorescence background
Pixels [Z dim]
Pixels [Y dim]
Pixels [X dim]
Please say that again
Output Directory
Choose
Output Stack Filename
Paint_HILO_Fluc
Show lasers
Show fluorophore spectra
Show PSF
Show data during acquisition Off On
Show photophysics statistics at end of simulation Off On
Use parallel computing
Force Overwrite
Launch simulation
Fluorescence sampling rate
Other general parameters
Launch ensemble simulation

b

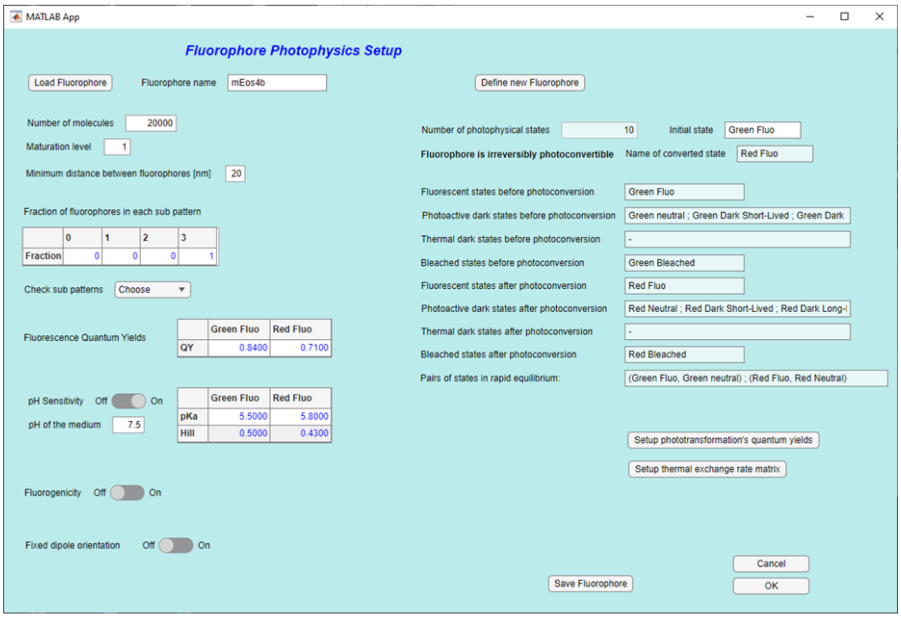
MATLAB App
Fluorophore Photophysics Setup
Load Fluorophore
Fluorophore name mEos4b
Define new Fluorophore
Number of molecules 20000
Maturation level 1
Minimum distance between fluorophores [nm] 20
Number of photophysical states 10
Initial state Green Fluo
Fluorophore is irreversibly photoconvertible
Name of converted state Red Fluo
Fluorescent states before photoconversion Green Fluo
Photoactive dark states before photoconversion Green neutral ; Green Dark Short-Lived ; Green Dark
Thermal dark states before photoconversion -
Bleached states before photoconversion Green Bleached
Fluorescent states after photoconversion Red Fluo
Photoactive dark states after photoconversion Red Neutral ; Red Dark Short-Lived ; Red Dark Long-l
Thermal dark states after photoconversion -
Bleached states after photoconversion Red Bleached
Pairs of states in rapid equilibrium: (Green Fluo, Green neutral) ; (Red Fluo, Red Neutral)
Fraction of fluorophores in each sub pattern
0 1 2 3
Fraction 0 0 0 1
Check sub patterns Choose
Fluorescence Quantum Yields
Green Fluo Red Fluo
QY 0.8400 0.7100
pH Sensitivity Off On
pH of the medium 7.5
Green Fluo Red Fluo
pKa 5.5000 5.8000
Hill 0.5000 0.4300
Setup phototransformation's quantum yields
Setup thermal exchange rate matrix
Fluorogenicity Off On
Fixed dipole orientation Off On
Cancel
Save Fluorophore
OK

c

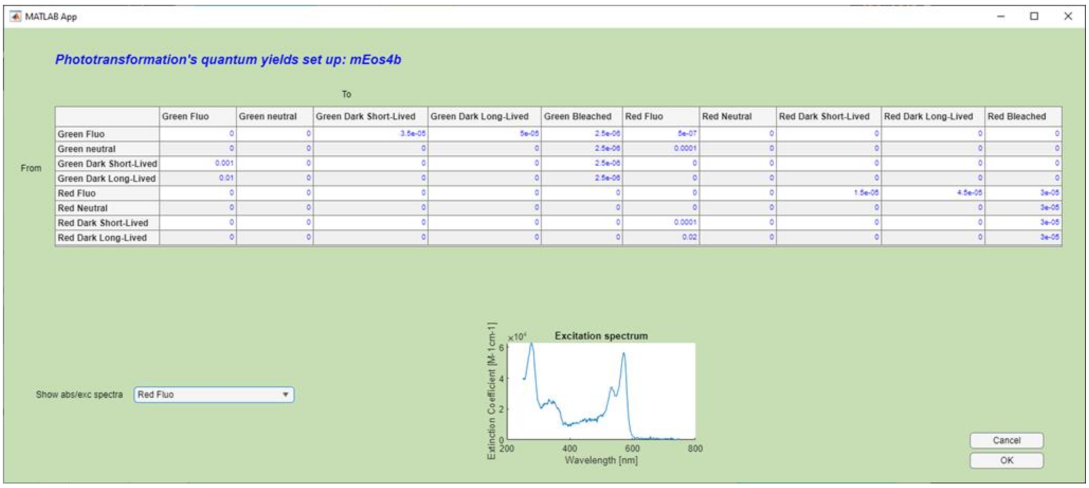
MATLAB App
Phototransformation's quantum yields set up: mEos4b
To
From
Green Fluo | Green neutral | Green Dark Short-Lived | Green Dark Long-Lived | Green Bleached | Red Fluo | Red Neutral | Red Dark Short-Lived | Red Dark Long-Lived | Red Bleached
Green Fluo 0 0 3.5e-05 5e-05 2.5e-06 5e-07 0 0 0 0
Green neutral 0 0 0 0 2.5e-06 0.0001 0 0 0 0
Green Dark Short-Lived 0.001 0 0 0 2.5e-06 0 0 0 0 0
Green Dark Long-Lived 0.01 0 0 0 2.5e-06 0 0 0 0 0
Red Fluo 0 0 0 0 0 0 0 1.5e-06 4.5e-05 3e-05
Red Neutral 0 0 0 0 0 0 0 0 0 3e-05
Red Dark Short-Lived 0 0 0 0 0 0.0001 0 0 0 3e-05
Red Dark Long-Lived 0 0 0 0 0 0.02 0 0 0 3e-05
Show abs/exc spectra Red Fluo
Excitation spectrum
Extinction Coefficient [M-1cm-1]
Wavelength [nm]
Cancel
OK

d

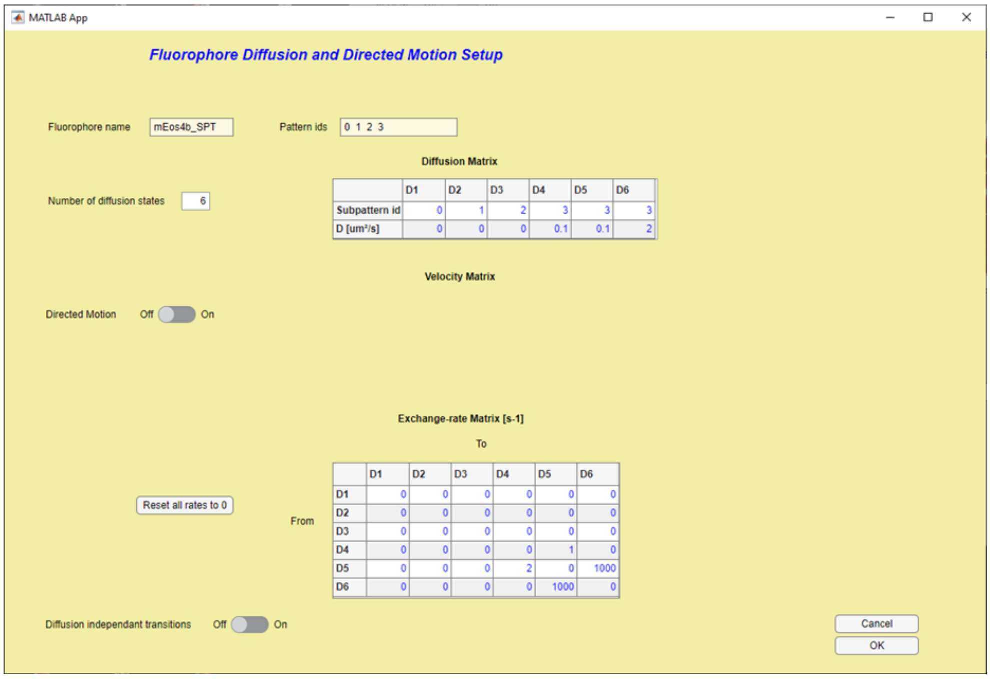

Supplementary Fig. S26

The SMIS graphical user interface. (a) Main SMIS panel. (b) Photophysical setup panel. (c) Phototransformation quantum yields setup panel. (d) SPT setup panel.

Supplementary References

1. Zitter, E. D. *et al.* Mechanistic investigation of mEos4b reveals a strategy to reduce track interruptions in sptPALM. *Nat Methods* **16**, 707–710 (2019).
2. Berardozzi, R., Adam, V., Martins, A. & Bourgeois, D. Arginine 66 Controls Dark-State Formation in Green-to-Red Photoconvertible Fluorescent Proteins. *J. Am. Chem. Soc.* **138**, 558–565 (2016).
3. Gidi, Y. *et al.* Unifying Mechanism for Thiol-Induced Photoswitching and Photostability of Cyanine Dyes. *J. Am. Chem. Soc.* **142**, 12681–12689 (2020).
4. Kasper, R., Heilemann, M., Tinnefeld, P. & Sauer, M. Towards ultra-stable fluorescent dyes for single-molecule spectroscopy. in *Biophotonics 2007: Optics in Life Science* vol. 6633 405–416 (SPIE, 2007).
5. Baibakov, M. & Wenger, J. Laser-induced fluorescence quenching of red fluorescent dyes with green excitation: Avoiding artifacts in PIE-FRET and FCCS analysis. *Chemical Physics Letters* **706**, 669–674 (2018).
6. Diekmann, R. *et al.* Optimizing imaging speed and excitation intensity for single-molecule localization microscopy. *Nat Methods* **17**, 909–912 (2020).
7. Venkataramani, V., Herrmannsdörfer, F., Heilemann, M. & Kuner, T. SuReSim: simulating localization microscopy experiments from ground truth models. *Nat Methods* **13**, 319–321 (2016).
8. Lindén, M., Ćurić, V., Boucharin, A., Fange, D. & Elf, J. Simulated single molecule microscopy with SMeagol. *Bioinformatics* **32**, 2394–2395 (2016).
9. Novák, T., Gajdos, T., Sinkó, J., Szabó, G. & Erdélyi, M. TestSTORM: Versatile simulator software for multimodal super-resolution localization fluorescence microscopy. *Sci Rep* **7**, 951 (2017).
10. Sage, D. *et al.* Super-resolution fight club: assessment of 2D and 3D single-molecule localization microscopy software. *Nat Methods* **16**, 387–395 (2019).
11. Lagardère, M., Chamma, I., Bouilhol, E., Nikolski, M. & Thoumine, O. FluoSim: simulator of single molecule dynamics for fluorescence live-cell and super-resolution imaging of membrane proteins. *Sci Rep* **10**, 19954 (2020).
12. Griffié, J. *et al. Virtual-SMLM, a virtual environment for real-time interactive SMLM acquisition*. 2020.03.05.967893 https://www.biorxiv.org/content/10.1101/2020.03.05.967893v1 (2020).
13. Heckert, A., Dahal, L., Tijan, R. & Darzacq, X. Recovering mixtures of fast-diffusing states from short single-particle trajectories. *eLife* **11**, e70169 (2022).

14. Adam, V. *et al.* Rational Control of Off-State Heterogeneity in a Photoswitchable Fluorescent Protein Provides Switching Contrast Enhancement**. *ChemPhysChem* **23**, e202200192 (2022).
15. Mantovanelli, A. M. R. *et al.* Photophysical studies at cryogenic temperature reveal a novel photoswitching mechanism of rsEGFP2. *bioRxiv* 2022.08.22.504779 (2022) doi:10.1101/2022.08.22.504779.
16. Subach, F. V. *et al.* Red fluorescent protein with reversibly photoswitchable absorbance for photochromic FRET. *Chemistry & biology* **17**, 745–55 (2010).
17. De Zitter, E. *et al.* Mechanistic Investigations of Green mEos4b Reveal a Dynamic Long-Lived Dark State. *J. Am. Chem. Soc.* **142**, 10978–10988 (2020).
18. Adam, V., Nienhaus, K., Bourgeois, D. & Nienhaus, G. U. Structural basis of enhanced photoconversion yield in green fluorescent protein-like protein Dendra2. *Biochemistry* **48**, 4905–4915 (2009).
19. Grimm, J. B. *et al.* Bright photoactivatable fluorophores for single-molecule imaging. *Nat. Methods* **13**, 985–988 (2016).
20. Kuhn, T., Hettich, J., Davtyan, R. & Gebhardt, J. C. M. Single molecule tracking and analysis framework including theory-predicted parameter settings. *Sci Rep* **11**, 9465 (2021).
