# Supplementary material for: Single molecule imaging simulations with advanced fluorophore photophysics
Source: Commun Biol. 2023 Jan 16;6:53. doi: 10.1038/s42003-023-04432-x (PMC9842740; doi:10.1038/s42003-023-04432-x)
Supplement: Supplementary file 3 — Description of Additional Supplementary Files [file 42003_2023_4432_MOESM3_ESM.pdf]

Description of Additional Supplementary Files

File name: Supplementary Software 1
Description: Source code, Matlab app, as well as freely usable standalone versions of SMIS vsn2.1 for Windows.

File name: Supplementary Data 1
Description: Source data for figures in Excel format.
